# ﻿Morphological and molecular data warrant the description of a new species of the genus *Scutiger* (Anura, Megophryidae) from the Central Himalaya

**DOI:** 10.3897/zookeys.1210.127106

**Published:** 2024-08-26

**Authors:** Sylvia Hofmann, Daniel Jablonski, Joachim Schmidt

**Affiliations:** 1 Leibniz Institute for the Analysis of Biodiversity Change, Museum Koenig, 53113 Bonn, Germany Leibniz Institute for the Analysis of Biodiversity Change, Museum Koenig Bonn Germany; 2 Helmholtz-Centre for Environmental Research GmbH – UFZ, Department Conservation Biology, 04318 Leipzig, Germany Helmholtz-Centre for Environmental Research GmbH – UFZ, Department Conservation Biology Leipzig Germany; 3 Department of Zoology, Comenius University in Bratislava, Bratislava, Slovakia Comenius University in Bratislava Bratislava Slovakia; 4 General and Systematic Zoology, Institute of Biosciences, University of Rostock, 18055 Rostock, Germany University of Rostock Rostock Germany

**Keywords:** Genetics, Himalaya, lazy toad, montane forests, morphology, taxonomy

## Abstract

Recent phylogenetic studies in Himalayan lazy toads of the genus *Scutiger* Theobald, 1868 revealed the presence of genetically deeply divergent lineages. The taxonomy of *S.nepalensis* sensu lato was re-assessed based on museum material considering molecular and morphological data. The results strongly support the recognition of a new species, *S.kanjiroba***sp. nov.** distributed along the Nepalese Kanjiroba massif. It is further shown that *S.sikimmensis* has an apparently much more restricted distribution range than previously thought. The frequent misidentification of *Scutiger* across large areas of the Himalaya-Tibet area highlights the significance of careful taxonomic evaluation of collection material and the need for the direct morphological comparison of closely related species when describing new species.

## ﻿Introduction

The Himalaya, recognised as one of the world’s biodiversity hotspots (Myers et al. 2000; Mittermeier et al. 2011), hosts a unique assemblage of flora and fauna, characterised by exceptional species richness and high levels of endemism. However, many species in this region remain taxonomically and biogeographically understudied, leading to knowledge gaps that hinder our understanding of fundamental aspects of the evolutionary history of Himalayan biota and the development of effective conservation strategies. Compounding these challenges is the escalating threat to Himalayan biodiversity posed by human activities such as deforestation, habitat fragmentation, poaching, and climate change (Pandit 2013; Paudel et al. 2018; Kattel 2022). Consequently, species and data collected from the Himalayan region hold immense conservation value and are crucial for unravelling broad-scale biodiversity patterns and their underlying mechanisms (e.g., Agarwal et al. 2014; Schmidt et al. 2016, 2023).

**Figure 1.**
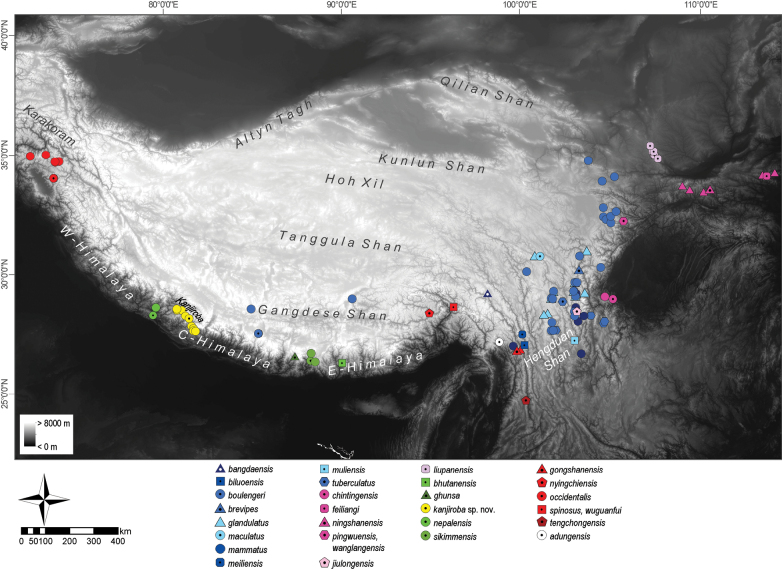
Overview of the Himalaya-Tibet orogenic area and occurrences of *Scutiger* species. Holotype localities are indicated by symbols with a central black dot. Some species lack the type locality, others could only be approximated due to imprecise information in the original description.

Lazy toads of the genus *Scutiger* Theobald, 1868, family Megophryidae, are endemic to the Himalaya-Tibet orogenic system and adapted to high montane and alpine areas. They occur from northern Pakistan, through Nepal, Bhutan, northern India, Myanmar, in the valleys of southern and eastern Tibet, and eastwards to southwestern China. According to Frost (2024), the genus comprises 29 recognised species, most of which distributed in the Hengduan Shan (Fig. 1). Only eight species are known from the Himalaya Mountain range: *S.occidentalis* Dubois, 1978 from its western part; *S.nepalensis* Dubois, 1974, *S.sikimmensis* (Blyth, 1855), and *S.ghunsa* Khatiwada, Shu, Subedi, Wang, Ohler, Cannatella, Xie, & Jiang, 2019 from the central Himalayan region; *S.bhutanensis* Delorme & Dubois, 2001, *S.nyingchiensis* Fei, 1977, *S.spinosus* Jiang, Wang, Li, & Che, 2016, and *S.wuguanfui* Jiang, Rao, Yuan, Wang, Li, Hou, Che, & Che, 2012 from the eastern part. Most of them show a disjunct distribution pattern which is hypothesised to result from the displacement of the species’ habitats from ancestral Tibet during the surface uplift of the Himalaya-Tibet orogen (HTO). The uplift-associated aridification of a warm temperate Miocene-Tibet, coupled with high extirpation rates of ancestral populations, and species range shifts along the drainage systems and epigenetic transverse valleys of the rising Himalaya explain the evolution of the present-day Himalayan *Scutiger* fauna most parsimoniously (Hofmann et al. 2024).

Interestingly, the assumed wide distribution range of the long-known species *S.nepalensis* differs considerably from the limited occurrences of other Himalayan *Scutiger* species. The type locality of this species is given as Khaptar [= Khaptad] area, in the Doti District of Chainpur in western Nepal. The species is assumed to occur in mountains between 3000 and 5000 m across a wide area of western Nepal (Jetz et al. 2012: https://mol.org/species/map/Scutiger_nepalensis). This large distribution contrasts with the above-mentioned biogeographic scenario that would lead us to expect a significant genetic structure and divergences with clear geographical pattern among the Himalayan *Scutiger* taxa. Recent advances in the phylogeography of *Scutiger* have revealed deeply divergent lineages with disjunct ranges within Himalayan taxa (Hofmann et al. 2017), necessitating a morphological and genetic re-evaluation of existing museum material considering the new phylogenetic findings. The presence of these genetically distinct lineages advocates the need for a taxonomic intervention, and we describe one of them as a new species herein, based on morphological and molecular evidence.

## ﻿Materials and methods

### ﻿Sampling

All voucher specimens and tissue samples for newly generated molecular data were obtained from museum holdings [Muséum national d’Histoire naturelle, Paris (**MNHN**); Natural history museum, Erfurt (**NHME**)] and had been collected in Nepal between 1973 and 1999. In total, the paratype series of *Scutigernepalensis* (*n* = 5), and four adult specimens, one subadult, and one tadpole individual of *S.nepalensis* sensu lato (s. l.) were investigated morphologically. For all these *S.nepalensis* s. l. we obtained molecular data. Additional DNA sequencing data of *S.nepalensis* s. l. were generated from tissue samples, available at the NHME, collected along with the voucher specimens by capture and release of individuals. The list of samples and their associated metadata are presented in Suppl. material 1: tables S1−S3.

### ﻿Laboratory work and molecular analysis

Total genomic DNA was isolated from tissues preserved in ethanol using the Qiagen DNeasy kit (Qiagen Inc.) following the manufacturer’s protocol. We amplified partial sequences of the following three mitochondrial (mt) and three nuclear (nu) loci via the polymerase chain reaction (PCR): 16S rRNA (550 bp), cytochrome oxidase subunit 1 (COI, 668 bp), and cytochrome b (Cytb, 985 bp), as well as beta-fibrinogen intron 7 (Bfib7, 508 bp), cyclin B2 gene intron 3 (Ccnb2, 777 bp), and recombination activating protein 1 gene (Rag1, 957 bp); for primers and PCR conditions see Hofmann et al. (2017). Heterozygotes in electropherograms of the nuclear loci were identified based on secondary peak calling. All protein coding gene fragments were translated into amino acids; no frameshift mutations or premature stop codons were observed. Nuclear alleles were not phased because most populations were represented by only a few or single individuals which did not allow a robust statistical inference of haplotypes. Therefore, polymorphic sites were encoded with the appropriate IUPAC ambiguity code. All newly generated sequences were deposited in GenBank (accession numbers: Suppl. material 1: tables S2, S3). Additional *Scutiger* and appropriate outgroup sequences corresponding to the six molecular target loci were retrieved from GenBank, combined with our new sequences, and subsequently aligned for each marker using MEGA11 software (Tamura et al. 2021).

DNA sequences from the three mitochondrial loci of each species were concatenated and used for Bayesian inference (BI) analysis, while the alignments of the three nuclear gene fragments were used separately for network analysis. Because several *Scutiger* species are represented in GenBank only by COI DNA sequence information, we additionally constructed a BI tree based on that single barcoding gene.

Phylogenetic trees were inferred with MrBayes using *Oreolalaxchuanbeiensis* Tian, 1983 and *O.omeimontis* (Liu & Hu, 1960) (concatenated mtDNA dataset), or *Oreolalaxxiangchengensis* Fei & Huang, 1983 (COI dataset) as outgroup. MrBayes was run for up to 5 million generations, sampling every 500^th^ generation. We used four parallel Markov chain Monte Carlo simulations with four chains and discarded the first 25% of the samples of each run as burn-in. Chain convergence was monitored with Tracer v. 1.7.1 (Rambaut et al. 2018). Phylogenetic networks were generated based on the nuDNA sequence data and uncorrected p-distances using the Neighbor-Net algorithm (Bryant and Moulton 2004) implemented in SplitsTree v. 4.19.2 (Huson and Bryant 2006). We also calculated genetic distances [uncorrected p-distance and based on maximum composite likelihood (MCL)] between species, and the mean genetic distance between samples within the new species using MEGA11.

### ﻿Morphological analysis

The following measurements were taken with dial callipers and recorded to the nearest 0.1 mm: Snout vent length (**SVL**), distance from tip of snout to posterior edge of vent; head length (**HL**), distance from angle of jaws to snout-tip; head width (**HW**), measured at posterior angle of jaws; snout length (**SL**), from tip of snout to anterior corner of eye; distance of naris to tip of snout (**NSD**); horizontal diameter of eye (**ED**); eyelid-naris distance (**END**), from naris to anterior edge of the eye; internarial distance (**IND**); forearm length (**FAL**), from flexed elbow to base of inner metacarpal tubercle; hand length (**HAL**), from base of inner metacarpal tubercle to tip of 3^rd^ finger; femur (thigh) length (**FEL**), from vent to outer edge of flexed knee; tibia (shank) length (**TIBL**), distance from outer edge of flexed knee to tip of heel; length of tarsus (**TaL**), distance from heel to proximal edge of inner metatarsal tubercle; foot length (**FOL**), distance from proximal edge of inner metatarsal tubercle to tip of 4^th^ toe. We further recorded the relative length of fingers and toes, presence/absence of subarticular tubercles, presence/absence of vocal sac, presence/absence of vomerine (**v**)/maxillary (**m**) dentation, presence/absence of tympanum, webbing of toes (developed, weak, rudimentary, absent), finger(s) with nuptial spines, presence/absence of pectoral (**p**)/axillary (**ax**) glands, gland size relation (p>>/>/= ax), presence/absence of spines on p/ax, presence/absence of spines on inner forearms, presence/absence of spines at belly, presence/absence of tubercles/warts on dorsal (and lateral) surfaces of body and/or limbs, presence/absence of black spines on dorsal tubercles. Sex was determined by the presence of nuptial spines on fingers and chest in males in breeding condition.

A Principal Component Analysis (PCA) was performed in R 4.3.3 (RCoreTeam 2024) using the packages FactoMineR (Le et al. 2008) and ggplot2 v. 3.5.0 (Wickham 2016). The PCA was based on metric data (values were normalised by SVL prior to PCA, except ratios) and included the unknown taxon (*Scutigernepalensis* s. l.) together with the phylogeographical closest species, namely *S.ghunsa* and *S.nepalensis*. For *S.sikimmensis*, comparable morphometric data for individual features were not available to us (see also Discussion). We further assessed SVL and the normalised metric characters for equal variances (F ≥ 0.05) across species via Levene’s test and analysed them by an analysis of variance (ANOVA) and TukeyHSD post hoc test. Because of the small sample size per group, we combined males and females for analysis.

The labial tooth row formula (**LTRF**) of the tadpole was recorded according to Dubois (1995), and developmental stage was determined following Gosner (1960).

### ﻿Comparative specimens

*Scutigernepalensis* Dubois, 1974 [1974.1096−1974.1098 and 1989.3361− 1989.3362, paratypes; one specimen of *S.nepalensis* (A1724/09); six specimens of *S.nepalensis* s. l. (A0570/99, A0574/99, A0576/99, A1248/05, A1249/05, one tadpole A1250/05) stored at the NHME]. Morphological data of other *Scutiger* species were extracted from original species descriptions and/or recompiled treatises, particularly (Fei et al. 2009, 2012).

All adult specimens were assigned to the genus *Scutiger* based on the following combination of features: toad-like morphology, tympanum, and tympanic ring entirely absent, pupil vertically elliptic, femoral glands indistinct, tongue rounded or slightly indented behind, fingers free, toes free or with some webbing, dorsum warty, pectoral (and axillary) glands and fingers with nuptial spines (in breeding males) (Myers and Leviton 1962; Fei et al. 2009; Fei and Ye 2016). Additional specimens had their species identity confirmed through molecular data.

## ﻿Results

### ﻿mtDNA analysis and phylogenetic networks of nuclear genes

**Figure 2.**
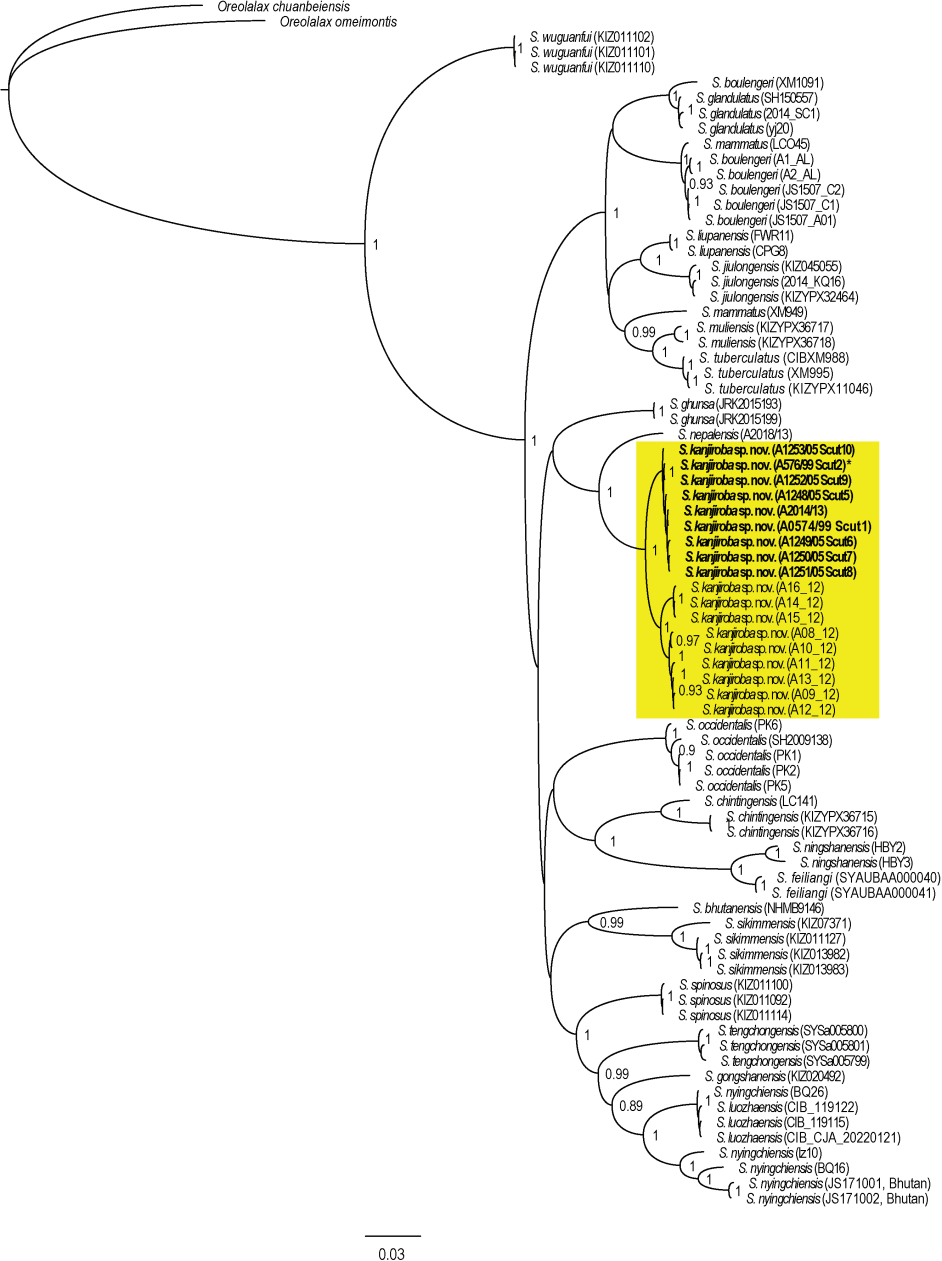
Bayesian inference tree based on the concatenated mtDNA sequence data of 16S, COI and Cytb. Node values are Bayesian posterior probabilities ≥ 0.7. Holotype (*) and paratype specimens are indicated bold. Only samples with data for more than one locus were included.

**Figure 3.**
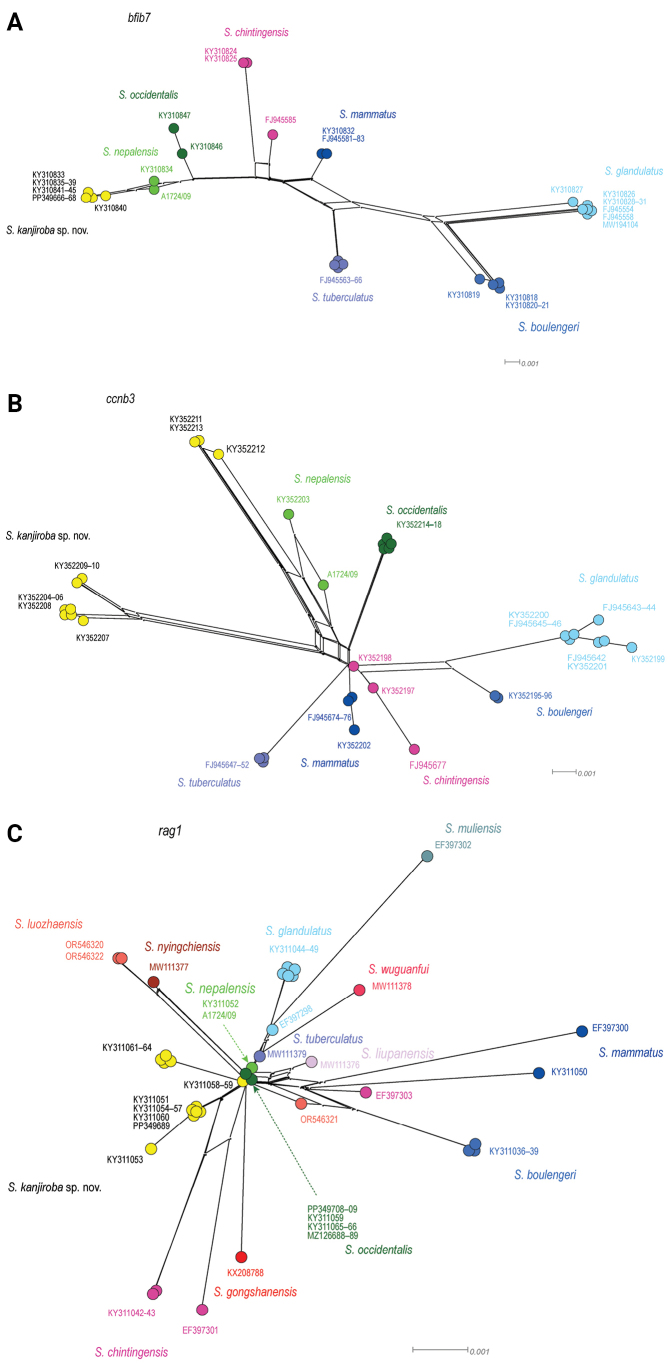
Neighbor-Net networks (Bryant and Moulton 2004) of the nuclear **A** Bfib7 **B** Ccnb2, and **C** Rag1 sequence information retrieved in *Scutiger*.

Phylogenetic trees of the mtDNA genes (Fig. 2, concatenated, 2203 bp: 16S, COI and Cytb; Suppl. material 1: fig. S1, 631bp: COI) and networks of the nuclear genes Bfib7, Ccnb2, and Rag1 (Fig. 3) recover specimens from the Kanjiroba Himal as distinct, deeply divergent sister clade of *S.nepalensis*, which is maximal supported in the tree from the concatenated data. The placement of the Kanjiroba/*S.nepalensis* clade in the tree might be discordant depending on the amount and type of molecular data (as is the case for the majority of known *Scutiger* taxa). However, its distinctness is robust and supported by COI uncorrected genetic distances to congeners which range between 7.4% and 15.5% (MCL: 5.9% and 13.5%). Noteworthy, the overall range of COI genetic distance between *Scutiger* species is from 2.4% to 16.7% (MCL: 1.9% to 14.4%; Suppl. material 1: tables S4, S5). Mean uncorrected p-distance within the Kanjiroba clade was 1.6% ± 0.3 (MCL: 1.6% ± 0.4).

Due to low quality and concentration of the DNA and resulting difficulties in amplification, the following specimens were only barcoded through 16S rRNA: for A0570/99 and A1254/05 we recovered > 99% identity and coverage to/of sequences of *S.kanjiroba* sp. nov., e.g., KY310787− KY310792 (Suppl. material 1: table S2), while A1724/09 (PP766266) matched unambiguously *S.nepalensis*.

### ﻿Morphological comparison

**Figure 4.**
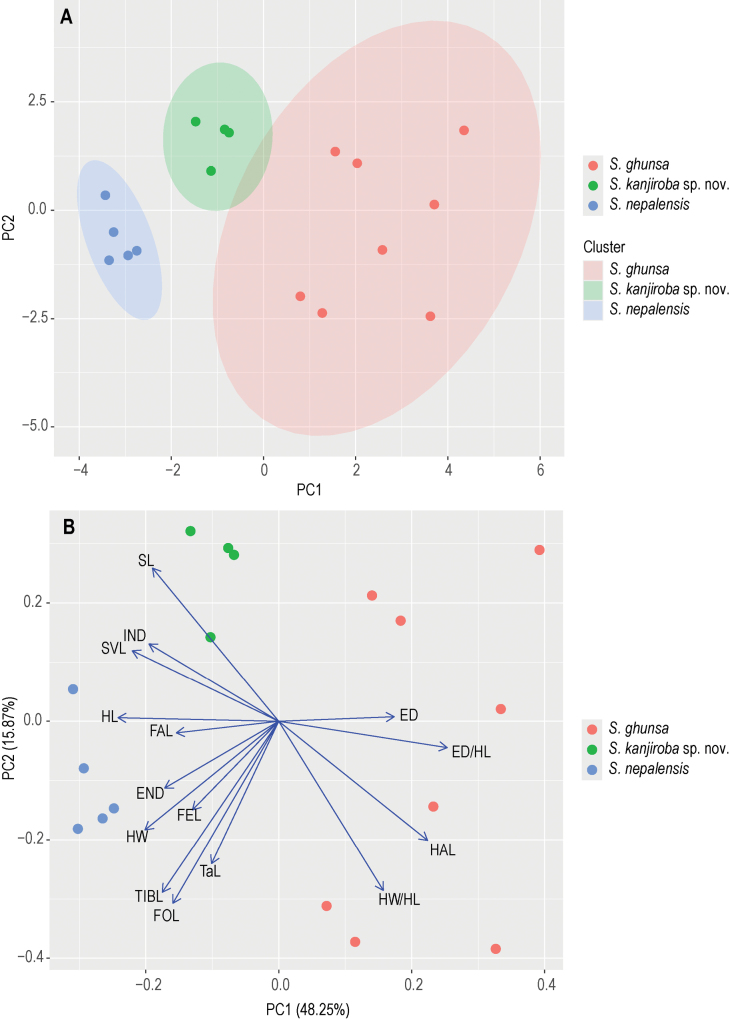
PCA of 15 metric variables in 17 adult specimens **A** with 95% confidence ellipses **B** with loadings. Abbreviations: SVL snout–vent length, HL head length; HW head width; SL snout length; ED horizontal diameter of eye; END eyelid-naris distance; IND internarial distance; FAL forearm length; HAL hand length; FEL femur (thigh) length; TIBL tibia (shank) length; TaL length of tarsus; FOL foot length.

The morphological comparisons between the *Scutiger* specimens from the Kanjiroba range and their congeners are comprehensively outlined in Suppl. material 1: table S5. Considering the geographic distribution of *Scutiger* species (Fig. 1) and our phylogenetic findings, the differentiation between the Kanjiroba lineage and *S.nepalensis*, *S.ghunsa*, and *S.sikimmensis*, holds significant relevance; all details of the morphological comparison among these taxa (except *S.sikimmensis*) are presented in Suppl. material 1: table S6. In the PCA analysis, specimens from the Kanjiroba range formed a distinct cluster, with the first component (PC1) explaining 48% of the data variance and the second (PC2) explaining 16% (Fig. 4). Subsequent ANOVA and TukeyHSD post hoc tests uncovered several statistically significant differences among the Kanjiroba lineage, *S.ghunsa*, and *S.nepalensis* (Suppl. material 1: fig. S2, table S7).

The *Scutiger* specimens from the Kanjiroba Himal can be readily distinguished from the geographically neighbouring *S.nepalensis* by a narrower and smaller head shape [HW/SVL: 0.34 (0.33−0.35) vs 0.40 (0.37−0.42); HL/SVL: 0.28 (0.27−0.29) vs 0.32 (0.30−0.34), and in a shorter foot length [FOL/SVL: 0.42 (0.41−0.43) vs 0.45 (0.40−0.47)] (Suppl. material 1: fig. S3, tables S6, S7). Of note, the head in *S.nepalensis* seems to be larger than in any other *Scutiger* species (Suppl. material 1: fig. S4). From other congeners the Kanjiroba lineage can be separated by a suite of morphological features as follows:

*S.adungensis* − the Kanjiroba lineage is distinguished by absence of vocal sac in adult males (vs presence); smaller body size (in males; SVL 55.4 mm vs 71.0−73.0 mm); the absence of maxillary teeth (vs presence); presence of numerous small, densely arranged spines on the surface of fingers I, II, and inner surface of finger III of males in breeding condition (vs large spines on inner two fingers); two pairs of spine patches on chest of breeding male (vs one pair).

*S.bangdaensis* − the Kanjiroba lineage is distinguished by rudimentary webbing between toes (vs developed), and larger body size (SVL: males 55.4 mm vs 45.5−50.0 mm; females 54.2−66.7 mm vs 48.0−49.5 mm).

*S.bhutanensis* − the Kanjiroba lineage is distinguished by presence of numerous small, densely arranged spines on surface of fingers I, II, and inner surface of finger III of males in breeding condition (vs large nuptial spines on first two fingers of males).

*S.biluoensis* − the Kanjiroba lineage is distinguished by absence of vomerine and maxillary teeth (vs presence); presence of nuptial spines on dorsal surface of fingers I, II, and inner surface of finger III of males in breeding condition (vs on inner two fingers); smaller male body size (SVL 55.4 mm vs 73.0 mm).

*S.boulengeri* − the Kanjiroba lineage is distinguished by rudimentary webbing between toes (vs developed); absence of spine patches on belly of males in breeding condition (vs presence).

*S.brevipes* − the Kanjiroba lineage is distinguished by rudimentary webbing between toes (vs developed); presence of nuptial spines on dorsal surface of fingers I, II, and inner surface of finger III of males in breeding condition (vs on inner two fingers).

*S.chintingensis* − the Kanjiroba lineage is distinguished by larger body size (SVL: males 55.4 mm vs 42.0−42.4 mm; females 54.2−66.7 mm vs 48.0−52.8 mm); absence of maxillary teeth (vs presence); rudimentary webbing between toes (vs developed, large or reduced); absence of spines on inner surface of upper arm and forearm of males in breeding condition (vs presence).

*S.feiliangi* − the Kanjiroba lineage is distinguished by larger body size (SVL: males 55.4 mm vs 45.7−50.2 mm; females 54.2−66.7 mm vs 48.9−51.5 mm); absence of spines on inner surface of forearm of males in breeding condition (vs presence); the absence of maxillary teeth (vs presence); absence of pectoral/axillary glands with spines in females in breeding condition (vs presence); absence of spines on belly of females in breeding condition (vs presence).

*S.ghunsa* − the Kanjiroba lineage is distinguished by larger body size (male: 55.4 mm vs 42.1−47.8 mm; female: 54.2−66.7 mm vs 50.2−53.9 mm); longer head [HL/SVL: 0.28 (0.27−0.29) vs 0.25 (0.24−0.26)]; shorter hand length [HAL/SVL: 0.25 (0.24−0.26) vs 0.46 (0.43−0.54)]; longer snout (SL/SVL male: 0.15 (0.14−0.15) vs 0.11 (0.10−0.12)]; relative length of toes 4>3>5>2>1 (vs 4>5>3>2>1); absence of tympanum (vs hidden tympanum); rudimentary webbing between toes (vs absence of webbing).

*S.glandulatus* − the Kanjiroba lineage is distinguished by smaller body size (SVL, male: 55.4 mm vs 67.0−81.0; female: 54.2−66.7 mm vs 77.0−81.0); relative length of toes 4>3>5>2>1 (vs 4>5>3>2>1); absence of subarticular tubercles (vs presence); rudimentary webbing between toes (vs developed); presence of nuptial spines on dorsal surface of fingers I, II, and inner surface of finger III of males in breeding condition (vs on inner two fingers); presence of spines on male axillary glands (vs absence).

*S.gongshanensis* − the Kanjiroba lineage is distinguished by absence of vocal sac in adult males (vs presence); absence of maxillary teeth (vs presence); presence of numerous small, densely arranged spines on the surface of fingers I, II, and inner surface of finger III of males in breeding condition (vs large spines on inner two fingers); rudimentary webbing between toes (vs absence of webbing); presence of a pair of pectoral and axillary patches in breeding male (vs one pair); presence of spine on tubercles on dorsum (vs absence);

*S.jiulongensis* − the Kanjiroba lineage is distinguished by smaller male body size (SVL, 55.4 mm vs 67.4−81.5 mm; absence of subarticular tubercles (vs presence); presence of nuptial spines on dorsal surface of fingers I, II, and inner surface of finger III of males in breeding condition (vs on inner two fingers).

*S.liupanensis* − the Kanjiroba lineage is distinguished by absence of maxillary teeth (vs presence); rudimentary webbing between toes (vs developed).

*S.luozhaensis* − the Kanjiroba lineage is distinguished by absence of more than one and up to six separated spines on top of each dorsal tubercle of males in breeding condition (vs presence of up to six spines on tubercles); absence of yellow tubercles scattered around cloaca of males in breeding condition (vs presence).

*S.maculatus* − the Kanjiroba lineage is distinguished by absence of subarticular tubercles (vs presence); rudimentary webbing between toes (vs developed).

*S.mammatus* − the Kanjiroba lineage is distinguished by absence of subarticular tubercles (vs presence); rudimentary webbing between toes (vs developed, large or reduced); presence of nuptial spines on dorsal surface of fingers I, II, and inner surface of finger III of males in breeding condition (vs on inner two fingers); presence of spines on male axillary glands (vs absence).

*S.meiliensis* − the Kanjiroba lineage is distinguished by absence of maxillary teeth (vs presence); presence of nuptial spines on dorsal surface of fingers I, II, and inner surface of finger III of males in breeding condition (vs on inner two fingers).

*S.muliensis* − the Kanjiroba lineage is distinguished by presence of nuptial spines on dorsal surface of fingers I, II, and inner surface of finger III of males in breeding condition (vs on inner two fingers); presence of a pair of pectoral and axillary patches in breeding male (vs one pair).

*S.ningshanensis* − the Kanjiroba lineage di is distinguished by absence of maxillary teeth (vs presence); absence of spine patches on belly of males in breeding condition (vs presence).

*S.nyingchiensis* − the Kanjiroba lineage is distinguished by absence of maxillary teeth (vs presence); rudimentary webbing between toes (vs developed).

*S.occidentalis* − the Kanjiroba lineage is distinguished by a smaller head (HL: 14.5−19.1 mm vs 23.5 mm; HW: 18.2−23.2 mm vs 24.5 mm); absence of spines on male forearm in breeding condition (vs presence); absence of dorsal tubercles with 1−6 coloured tips (corresponding to spines in breeding condition) on top (vs presence, Suppl. material 1: fig. S5); rudimentary webbing between toes (vs developed).

*S.pingwuensis* − the Kanjiroba lineage is distinguished by absence of spines on male forearm in breeding condition (vs presence); absence of spine patches on belly of males in breeding condition (vs presence).

*S.sikimmensis* − the Kanjiroba lineage is distinguished by relative length of toes 4>3>5>2>1 (vs 4>5>3>2>1); presence of spines on dorsal tubercles of males (vs absence); see also Suppl. material 1: fig. S6 for syntypes of *S.sikimmensis* and Discussion.

*S.spinosus* − the Kanjiroba lineage is distinguished by absence of spines on male upper arm in breeding condition (vs presence); absence of spines on dorsal tubercles of females (vs presence); wider internarial distance (5.6−6.8 mm vs 4.0−4.8 mm).

*S.tengchongensis* − the Kanjiroba lineage is distinguished by larger male body size (SVL: 55.4 mm vs 36.0−40.1 mm); absence of small patches of black spines near armpit of males in breeding condition (vs presence).

*S.tuberculatus* − the Kanjiroba lineage is distinguished by smaller male body size (SVL: 55.4 mm vs 68.0−76.0 mm); presence of nuptial spines on dorsal surface of fingers I, II, and inner surface of finger III of males in breeding condition (vs on inner two fingers).

*S.wanglangensis* − the Kanjiroba lineage is distinguished by absence of maxillary teeth (vs presence); absence of spine patches on belly of males in breeding condition (vs presence).

*S.wuguanfui* − the Kanjiroba lineage is distinguished by absence of vocal sac in adult males (vs presence); absence of small black spines on upper chest (vs presence).

### ﻿Taxonomy

Based on the synthesis of molecular phylogenetic findings and observed morphological distinctions, the *Scutiger* populations inhabiting the Kanjiroba Himal region in western Nepal are deemed to constitute a novel species. Consequently, we provide a detailed description of this species.

#### ﻿ Scutiger kanjiroba sp. nov.

Taxon classificationAnimaliaAnuraMegophryidae

B7133399-1BE2-5E99-897C-955D6E7DB6A5

https://zoobank.org/5C6D6A08-E956-43C6-B2E8-3F505AC8F38A

**Figure 5.**
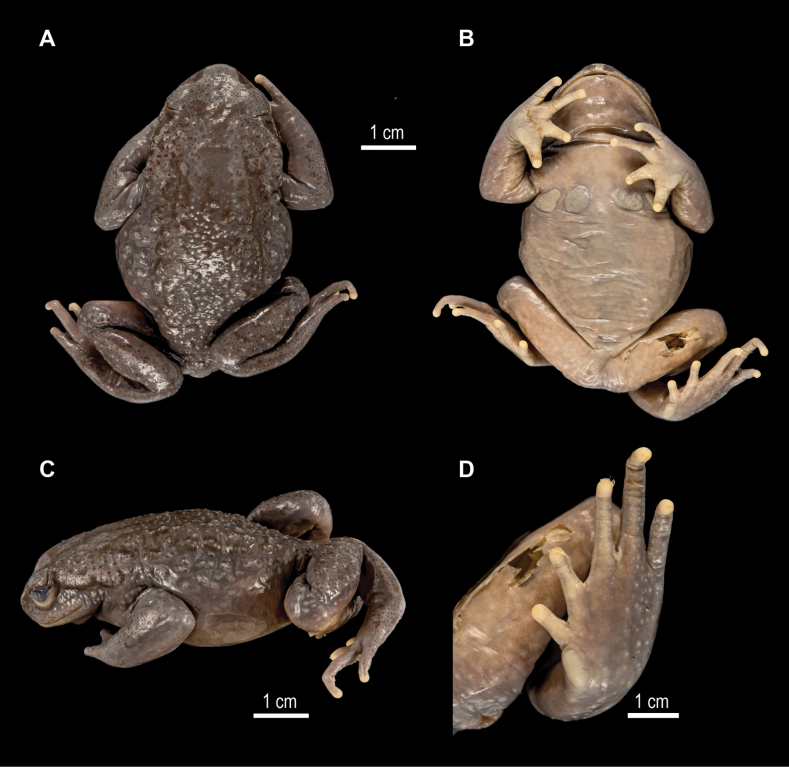
*Scutigerkanjiroba* sp. nov. holotype NHME A0576/99 in preservative (credit: Morris Flecks) **A** dorsal view of body **B** ventral view **C** dorsolateral view of body **D** ventral view of left foot.

Fig. 5 ; Suppl. material 1: figs S3, S7, tables S5, S6 Suggested common name: Kanjiroba lazy toad

Scutiger nepalensis : Hofmann et al. (2017), partim.

##### Type material.

***Holotype.*** Nepal • 1 ♂; NHME A0576/99; adult; collected at Juphal in the direction to Tripurakot, Dolpa district, Nepal (29°01'N, 82°47'E, 2600 m a.s.l.) on 31/05/1997 by Marco Fischer; deposited in the NHME. ***Paratypes***. Nepal • 1 ♀; NHME A0570/99; adult; and 1 juvenile; NHME A1247/05; collected at Talphi, Jumla district, Nepal (29°18'N, 82°20'E, 3400 m a.s.l.) on 16/06/1997 by Marco Fischer; deposited in the NHME. 1 ♀; NHME A0574/99; adult; collected at Kaigaon, Jumla district, Nepal (29°06'N, 82°35'E, 3600 m a.s.l.) on 03/06/1997 by Marco Fischer; deposited in the NHME. 1 ♀; NHME A1248/05, adult, collected at Dhauli Lake, Jumla district, Nepal (29°21'N, 82°23'E, 4400 m a.s.l.) on 05/07/1999 by Marco Fischer, deposited in the NHME. 1 subadult; NHME A1249/05; collected at Dhauli Lake, Jumla district, Nepal (29°21'N, 82°23'E, 4400 m a.s.l.) on 05/07/1999 by Marco Fischer, deposited in the NHME. 1 tadpole; NHME A1250/05; collected at Dhauli Lake, Jumla district, Nepal (29°21'N, 82°23'E, 4400 m a.s.l.) on 05/07/1999 by Marco Fischer, deposited in the NHME. 2 juveniles; NHME A1251/05 and A1252/05; collected at Dhauli Lake, Jumla district, Nepal (29°21'N, 82°23'E, 4400 m a.s.l.) on 05/07/1999 by Marco Fischer, deposited in the NHME. 1 juvenile; NHME A1253/05; collected at Bumra [Bumramadichaur], Jumla district, Nepal (29°23'N, 82°08'E) on 22/06/1999 by Ulrich Scheidt, deposited in the NHME. 1 juvenile; NHME A1254/05; collected at Maharigaon, Jumla district, Nepal (29°20'N, 82°23'E) on 08/07/1999 by Andreas Weigel, deposited in the NHME. 1 metamorp; NHME A2014/13; collected near Khari La, Jumla district, Nepal (29°21'N, 82°09'E, 3285 m a.s.l.) in 1999 by Marco Fischer, deposited in the NHME.

##### Diagnosis.

*Scutigerkanjiroba* sp. nov. is assigned to the genus *Scutiger* based in the above-mentioned morphological features and by its molecular phylogenetic position relative to other *Scutiger* species. It can be separated from all other species of the genus *Scutiger* by the combination of the following characters (for character states in other species see section Morphological comparison, above): (1) moderate body size, SVL adult male 55.4 mm (*n* = 1), adult female 54.2−66.7 mm (*n* = 3); (2) vomerine and maxillary dentation absent; (3) vocal sac absent; (4) subarticular tubercles absent; (5) presence of numerous small, densely arranged spines on the surface of fingers I, II and inner surface of finger III of males in breeding condition; (6) relative length of fingers 3>4>2>1; (7) spines on (inner) (fore)arms absent; (8) toes rudimentary webbed; (9) relative length of toes 4>3>5>2>1; (10) a pair of pectoral glands and a pair of axillary glands; (11) spines on pectoral and axillary glands present in males; (12) belly without spines; (13) tubercles/warts on dorsal and lateral surfaces of body present; (14) dorsal tubercles with black spines of males in breeding condition.

##### Description of holotype.

(Fig. 5): Measurements are provided in Suppl. material 1: table S6; all morphological measurements in mm. Adult male SVL 55.4; head moderate, wider than length (HW/HL 1.3); snout short (SL 7.9) and rounded, slightly protruding beyond jaw; canthus rostralis obvious; nostril dorsolateral, closer to tip of snout than eyes (SND 3.4, END 4.0); loreal region slightly oblique and concave; internarial distance larger than distance from anterior margin of eye to nostril (IND/END 1.4); eye moderately in size (EHD/HL 0.4); pupil vertical; interorbital space flat; tympanum and tympanic ring absent; supratympanic ridge moderately thick, from posterior part of upper eyelids to shoulder; tongue oval; choanae oval, widely separated, visible when viewed from below; vomerine and maxillary teeth absent; vocal sac absent.

Forelimbs strong; forearm of median length (FAL/SVL 0.25) and of similar length as the hand (HAL/SVL 0.24), without spines; fingers slender, without webbing and lateral dermal fringes; all fingertips rounded, not dilated; relative finger lengths: 3>4>2>1; subarticular tubercles absent; inner metacarpal tubercle flat and distinct, lager than the outer, indistinct metacarpal tubercle; nuptial spines on dorsal and lateral surface of first and second fingers, and on inner side of third finger.

Hindlimbs robust, moderate in length (TIBL/SVL = 0.38), thighs slightly shorter than shanks FEL/TIBL = 1.1), heels do not touch when folded at right angles to the body (see also Fig. 5A); foot longer than shank (FOL/TIBL = 1.1); tips of toes round; toes not webbed and without lateral fringes; toes relative lengths 4>3>5>2>1; subarticular tubercles absent; moderately large inner metatarsal tubercle, outer metatarsal tubercle indistinct.

Dorsal and lateral skin with distinct tubercles in preservative, each tubercle with creamy coloured keratinised tip; tubercles with small, keratinised tips present below and on supratympanic fold and on shank; forehead and surfaces of lower arm relatively smooth; throat and belly surface smooth; a pair of pectoral glands and a pair of axillary glands present on chest, pectoral glands only slightly larger than axillary glands, all covered by tiny spines.

##### Sexual dimorphism.

In males, a pair of pectoral glands and a pair of axillary glands present on breast (absent in females), all of them covered by spines in breeding season.

##### Colour in preservative.

The background colouration of the dorsum fades to grey, tubercles with creamy white point, dorsal surface of finger I, II and inner surface of finger III pale brown; ventral belly pale greyish; ventral limbs, ventral chest, and ventral head greyish pale brown.

##### Tadpole.

One tadpole (NHME A1250/05) of *Scutigerkanjiroba* sp. nov. was collected at stage 36 (Gosner, 1960) (Suppl. material 1: fig. S7); LTRF: 1:2+2/2+2:1.

##### Distribution and ecological notes.

*Scutigerkanjiroba* is a forest-dwelling species that is currently known from high-montane areas of the Kanjiroba Himal at altitudes between ~ 3300 and 4400 m a.s.l. in west Nepal (Fig. 1).

##### Etymology.

The specific epithet *kanjiroba* is a geographical proper name referring to the Kanjiroba Himal in Nepal. The southern area of the Kanjiroba Himal is so far the only known occurrence region of this new species.

## ﻿Discussion

Lazy toads of the genus *Scutiger* are a characteristic faunal element of the Himalaya-Tibet orogen. These toads possess limited dispersal capabilities, are adapted to high altitudes, and are typically found throughout the Himalaya Mountain range, exhibiting allopatric distribution with a high degree of local endemism. Previous research has revealed extensive geographically structured relationships among Himalayan *Scutiger* species, indicating significant physical barriers not only between species but also among populations (Hofmann et al. 2017). As taxonomic efforts intensify, we anticipate more frequent re-evaluation of *Scutiger* populations and taxonomic descriptions across the Himalaya. Ideally, this process will yield precise taxonomic conclusions and (hopefully) contribute to resolving the taxonomic confusion within this group.

The newly identified species *Scutigerkanjiroba* sp. nov. is phylogenetically distinct from the geographically neighbouring *S.nepalensis* Dubois, 1974, forming its sister lineage, as confirmed by both morphology and nuclear molecular analysis. It can be easily distinguished from *S.nepalensis* in the field by a much smaller and narrower head and an overall smaller body size. The two species are geographically separated by the Karnali River gorge, which probably acts as physical barrier to dispersal for terrestrial, non-flying organism groups.

The other species long known from the Central Himalaya, *S.sikimmensis* (Blyth, 1855), lacks a specified type locality in Sikkim. A neotype (BMNH 1887.11.2.15) had been assigned by Dubois (Dubois 1987 “1986”) from Yak La, Sikkim, but was set aside by the discovery of two syntypes ZSIC 9854−55 which originated from the Sikkim area (see Suppl. material 1: fig. S6). The (invalid) neotype is separated by a straight-line distance of <20 km from vouchers recently sampled in Yadong, Tibet, China. The latter had been sequenced and identified as *S.sikimmensis*. The *S.kanjiroba* sp. nov. populations from west Nepal do not share a common distribution area with *S.sikimmensis* and do not form a common phylogenetic clade with this species. Additionally, *S.ghunsa* Khatiwada, Shu, Subedi, Wang, Ohler, Cannatella, Xie & Jiang, 2019 is geographically situated between *S.kanjiroba* sp. nov. and *S.sikimmensis*. This implies that even if the exact location of *S.sikimmensis* is not precisely known, it can be assumed that *S.kanjiroba* sp. nov. represents a different species, which can be clearly distinguished morphologically and at the molecular level from *S.sikimmensis*. It further implicates that the geographic range of *S.sikimmensis* is vastly overestimated, e.g., by the International Union for Conservation of Nature (IUCN) Red List, scientific web applications like Map of Life (Jetz et al. 2012), and in the pertinent literature (e.g., Schleich and Kästle 2002). We assume that the distributional area of *S.sikimmensis* is mainly restricted to the Chumbi valley, with temperate climate, at the intersection of India (Sikkim), Bhutan, and China (Tibet) in the eastern part of the Himalaya (Suppl. material 1: fig. S8). The Chumbi valley corresponds largely to the administrative unit Yadong County in the Tibet Autonomous Region of China and is separated by the Dongkya (Chola) Mountain range from the southeastern frontier of Sikkim; the Dongkya range is cut by several passes, e.g., Cho La, Yak La, Nathu La, and Jelep La.

The discovery of *S.kanjiroba* sp. nov. in the Kanjiroba massif, west Nepal, adds to the recent description of *S.ghunsa* in east Nepal (Khatiwada et al. 2019), underscoring the high degree of amphibian diversity in Himalayan cloud forests and the conservation importance of these habitats.

## Supplementary Material

XML Treatment for Scutiger kanjiroba

## ﻿Additional information

### Author ORCIDs

Sylvia Hofmann https://orcid.org/0000-0003-0621-9049

Daniel Jablonski https://orcid.org/0000-0002-5394-0114

Joachim Schmidt https://orcid.org/0000-0002-0247-5208

### Data availability

All of the data that support the findings of this study are available in the main text or Supplementary Information.

## Supplementary materials

Supplementary material 1 Supplementary information Data type pdf https://binary.pensoft.net/file/1120960This dataset is made available under the Open Database License (http://opendatacommons.org/licenses/odbl/1.0/). The Open Database License (ODbL) is a license agreement intended to allow users to freely share, modify, and use this Dataset while maintaining this same freedom for others, provided that the original source and author(s) are credited.Sylvia Hofmann, Daniel Jablonski, Joachim Schmidt

## References

[B1] Agarwal I Bauer AM Jackman TR Karanth KP (2014) Insights into Himalayan biogeography from geckos: A molecular phylogeny of *Cyrtodactylus* (Squamata: Gekkonidae). Molecular Phylogenetics and Evolution 80: 145–155. 10.1016/j.ympev.2014.07.018 25108260

[B2] Bryant D Moulton V (2004) Neighbor-net: An agglomerative method for the construction of phylogenetic networks. Molecular Biology and Evolution 21(2): 255–265. 10.1093/molbev/msh018 14660700

[B3] Dubois A (1987) Miscellanea taxinomica batrachologica (I). Alytes 5: 7–95 (1986). https://www.biodiversitylibrary.org/page/62924166#page/9/mode/1up

[B4] Dubois A (1995) Keratodont formulae in anuran tadpoles: Proposal for standardisation. Journal of Zoological Systematics and Evolutionary Research 33: 1–15. 10.1111/j.1439-0469.1995.tb00207.x

[B5] Fei L Ye C-y (2016) Amphibians of China, Volume 1. Chengdu Institute of Biology, Chinese Academy of Sciences, Science Press, Beijing, China, 461 pp.

[B6] Fei L Hu S-q Huang Y-z (2009) Fauna Sinica. Amphibia. Volume 2. Anura. Chinese Academy of Science. Science Press, Beijing, 957 pp.

[B7] Fei L Ye CJ Jiang JP (2012) Colored Atlas of Chinese Amphibians and Their Distributions. Sichuan Science and Technology Press, Sichuan, China, 620 pp.

[B8] Frost DR (2024) Amphibian species of the world: an online reference. Version 6.2. https://amphibiansoftheworld.amnh.org/index.php [accessed 19 May 2024]

[B9] Gosner KL (1960) A simplified table for staging anuran embryos and larvae with notes on identification. Herpetologica 16: 183–190. https://www.jstor.org/stable/3890061

[B10] Hofmann S Stoeck M Zheng Y Ficetola FG Li JT Scheidt U Schmidt J (2017) Molecular Phylogenies indicate a Paleo-Tibetan Origin of Himalayan Lazy Toads (*Scutiger*). Scientific Reports 7(1): 3308. 10.1038/s41598-017-03395-4 28607415 PMC5468327

[B11] Hofmann S Podsiadlowski L Andermann T Matschiner M Baniya CB Litvinchuk SN Martin S Masroor R Yang J Zheng Y Jablonski D Schmidt J (2024) The last of their kind: Is the genus *Scutiger* (Anura: Megophryidae) a relict element of the paleo-transhimalaya biota? Molecular Phylogenetics and Evolution 2024: 108166. 10.1016/j.ympev.2024.108166 39127262

[B12] Huson DH Bryant D (2006) Application of phylogenetic networks in evolutionary studies. Molecular Biology and Evolution 23(2): 254–267. 10.1093/molbev/msj030 16221896

[B13] Jetz W McPherson JM Guralnick RP (2012) Integrating biodiversity distribution knowledge: Toward a global map of life. Trends in Ecology & Evolution 27(3): 151–159. 10.1016/j.tree.2011.09.007 22019413

[B14] Kattel GR (2022) Climate warming in the Himalayas threatens biodiversity, ecosystem functioning and ecosystem services in the 21^st^ century: Is there a better solution? Biodiversity and Conservation 31(8–9): 2017–2044. 10.1007/s10531-022-02417-6

[B15] Khatiwada JR Shu GC Subedi TR Wang B Ohler A Canatella DC Xie F Jiang JP (2019) A new species of megophryid frog of the genus *Scutiger* from Kangchenjunga Conservation Area, eastern Nepal. Asian Herpetological Research 10: 139–157. https://archive.org/details/ahr-20190301

[B16] Le S Josse J Husson F (2008) FactoMineR: An R Package for Multivariate Analysis. Journal of Statistical Software 25(1): 1–18. 10.18637/jss.v025.i01

[B17] Mittermeier RA Turner WR Larsen FW Brooks TM Gascon C (2011) Global Biodiversity Conservation: The Critical Role of Hotspots In: Zachos FE Habel JC (Eds) Biodiversity Hotspots.Springer, Berlin / Heidelberg, 3–22. 10.1007/978-3-642-20992-5_1

[B18] Myers GS Leviton AE (1962) Generic Classification of the High-Altitude Pelobatid Toads of Asia (*Scutiger*, *Aelurophryne*, and *Oreolalax*). Copeia 1962(2): 287–291. 10.2307/1440892

[B19] Myers N Mittermeier RA Mittermeier CG da Fonseca GA Kent J (2000) Biodiversity hotspots for conservation priorities. Nature 403(6772): 853–858. 10.1038/35002501 10706275

[B20] Pandit MK (2013) The Himalayas must be protected. Nature 501(7467): 283. 10.1038/501283a 24048033

[B21] Paudel PK Sipos J Brodie JF (2018) Threatened species richness along a Himalayan elevational gradient: Quantifying the influences of human population density, range size, and geometric constraints. BMC Ecology 18(1): 6. 10.1186/s12898-018-0162-3 29415707 PMC5803900

[B22] Rambaut A Drummond AJ Xie D Baele G Suchard MA (2018) Posterior Summarization in Bayesian Phylogenetics Using Tracer 1.7. Systematic Biology 67(5): 901–904. 10.1093/sysbio/syy032 29718447 PMC6101584

[B23] RCoreTeam (2024) R: A language and environment for statistical computing. R Foundation for Statistical Computing, Vienna, Austria. https://www.R-project.org [accessed 26 April 2024]

[B24] Schleich HH Kästle W (2002) Amphibians and Reptiles of Nepal. A.R.G. Gantner Verlag, Ruggell, 1200 pp.

[B25] Schmidt J Opgenoorth L Miehe G (2016) Speciation, uplift, and climate change. In: Miehe G Pendry C (Eds) Nepal.An introduction to the natural history, ecology and human environment in the Himalayas. A companion to the Flora of Nepal. Royal Botanic Garden Edinburgh, Edinburgh, 168–173.

[B26] Schmidt J Opgenoorth L Mao K Baniya CB Hofmann S (2023) Molecular phylogeny of mega-diverse *Carabus* attests late Miocene evolution of alpine environments in the Himalayan-Tibetan Orogen. Scientific Reports 13(1): 13272. 10.1038/s41598-023-38999-6 37582802 PMC10427656

[B27] Tamura K Stecher G Kumar S (2021) MEGA11: Molecular Evolutionary Genetics Analysis Version 11. Molecular Biology and Evolution 38(7): 3022–3027. 10.1093/molbev/msab120 33892491 PMC8233496

[B28] Wickham H (2016) ggplot2: Elegant Graphics for Data Analysis. Springer-Verlag, New York, 260 pp. https://link.springer.com/chapter/10.1007/978-3-319-24277-4_9

