# Supplementary material for: ﻿Morphological and molecular data warrant the description of a new species of the genus Scutiger (Anura, Megophryidae) from the Central Himalaya
Source: Zookeys. 2024 Aug 26;1210:229–46. doi: 10.3897/zookeys.1210.127106 (PMC11369495; doi:10.3897/zookeys.1210.127106)
Supplement: Supplementary material 1 — Supplementary information [file zookeys-1210-229_article-127106__-s001.pdf]

Supplementary Information

Morphological and molecular data warrant the description of a new species of the genus *Scutiger* (Anura, Megophryidae) from the Central Himalaya

Sylvia Hofmann, Daniel Jablonski, Joachim Schmidt

Table S1. List of *Scutiger* specimens used in this study for morphological investigation. §Holotype: *S. kanjiroba* sp. nov. A0576/99 (lab ID Scut2); *paratype. Specimens for which molecular data have been collected are indicated by italicized IDs. MNHN: Muséum national d'Histoire naturelle, Paris, France; NHME: Natural History Museum Erfurt, Germany; f: female, m: male, sa: subadult.

Source	ID	*Scutiger* species	locality	lat	long	age	sex
MNHN	1974.1096*	*nepalensis*	Chainpur, 20 km à l'ouest du lac sacré de Dah Khaptar [Chainpur, 20 km west of the sacred Dah Khaptad lake]	29.30	80.99	adult	m
MNHN	1974.1097*	*nepalensis*	Chainpur, 20 km à l'ouest du lac sacré de Dah Khaptar	29.30	80.99	adult	f
MNHN	1974.1098*	*nepalensis*	Chainpur, 20 km à l'ouest du lac sacré de Dah Khaptar	29.30	80.99	adult	f
MNHN	1989.3361*	*nepalensis*	Chainpur, 20 km à l'ouest du lac sacré de Dah Khaptar	29.30	80.99	adult	m
MNHN	1989.3362*	*nepalensis*	Chainpur, 20 km à l'ouest du lac sacré de Dah Khaptar	29.30	80.99	adult	m
NHME	*A1724/09*	*nepalensis*	Nepal, Seti, Doti, Chainpur; 20 km SSW, Khaptad-NP, Tribeni, Lukause Khola, 3000 m NN, river valley	29.37	81.14	adult	f
NHME	*A0570/99**	*kanjiroba* **sp. nov.**	Nepal, Thalpi	29.30	82.34	adult	f
NHME	*A0574/99**	*kanjiroba* **sp. nov.**	Nepal, Kaigaon	29.10	82.58	adult	f
NHME	*A0576/99*§	*kanjiroba* **sp. nov.**	Nepal, Dolpa District	29.02	82.79	adult	m
NHME	*A1248/05**	*kanjiroba* **sp. nov.**	Nepal, Dhauli Lake, 25 km NE Jumla	29.36	82.40	adult	f
NHME	*A1249/05**	*kanjiroba* **sp. nov.**	Nepal, Dhauli Lake, 25 km NE Jumla	29.36	82.40	sa	

Table S2. List of samples used in this study for phylogenetic reconstruction. Accession numbers of sequences generated in this study are indicated by grey cells. §Holotype: *S. kanjiroba* **sp. nov.** A0576/99 (lab ID Scut2); *paratype. #barcoded only.

Scutiger species	*Voucher/sample*	*Locality*	*Lat*	*Long*	*Alt*	*16S*	*coI*	*cytb*
bhutanensis	NHMB9146	Bhutan	27°24'	90°08'		PP344605	PP355376	–
boulengeri	KIZ08195	China, Tibet				–	JN700837	–
boulengeri	KIZ06712	China, Tibet, Bomi	29°51'	95°46'	3650	–	KU243064	–
boulengeri	KQ16_14	China, Sichuan	30°22'	101°40'	4062	KY310759	KY310869	KY310921
boulengeri	A1_AL	China, Tibet	29°36'	85°44'	5067	KY310760	KY310870	KY310922
boulengeri	A2_AL	China, Tibet	29°36'	85°44'	5067	KY310761	KY310871	KY310923
boulengeri	JS1507_C1	China, Tibet	30°19'	91°31'	4400	KY310765	KY310875	KY310927
boulengeri	JS1507_C2	China, Tibet	30°19'	91°31'	4400	KY310766	KY310876	KY310928
boulengeri	JS1507_A01	China, Tibet	30°36'	91°30'	4650	PP344606	PP355377	PP349710
boulengeri	XM1091	China, Tibet, Nyingchi	29°40'	94°42'	3850	KY310767	–	KY310929
chintingensis	KIZYPX36715					–	MW021352	MW133414
chintingensis	KIZYPX36716					–	MW021353	MW133415
chintingensis	LC141 01_6/01_9	China, Sichuan, Tianquan	30°14'	102°30'	2500	KY310769	KY310878	KY310930
feiliangi	SYAUBAA000040	China, Henan, Luoyang	34°41'	112°26'		–	OR263444	OR257694
feiliangi	SYAUBAA000041	China, Henan, Luoyang	34°41'	112°26'		–	OR263445	OR257695
ghunsa	JRK2015_193	Nepal, Taplejung, Ghunsa	27°39'	87°56'	3740	–	MK970591	MK970614
ghunsa	JRK2015_199	Nepal, Taplejung, Ghunsa	27°39'	87°56'	3740	–	MK970597	MK970620
glandulatus	2014_Sc1	China, Sichuan, Garzê	31°40'	99°43'	3457	KY310770	KY310879	KY310931
glandulatus	SH150557	China, Sichuan, Garzê	29°47'	100°25'	3713	KY310774	KY310883	KY310935
glandulatus	YJ20					OM505466	OM506149	OM505263
gongshanensis	CIB20070717001	China, Yunnan, Gongshan	25°45'	103°16'	2600	–	KU243062	–
gongshanensis	CIB20070717002	China, Yunnan, Gongshan	25°45'	103°16'	2600	–	KU243063	–
gongshanensis	KIZ020492					–	MW021395	MW133458
jiulongensis	KIZ045055	China, Sichuan, Ganzi	30°4'	101°57'		–	KU243066	MW133417
jiulongensis	KIZYPX32464					–	MW021354	MW133416
kanjiroba **sp. nov.**	A2014/13*	Nepal, Jumla District	29°21'	82°09'	3285	KY310776	KY310885	KY310936
kanjiroba **sp. nov.**	A8_12	Nepal, Rukum District	28°42'	82°55'	3340	KY310778	KY310887	KY310938
kanjiroba **sp. nov.**	A9_12	Nepal, Baglung District	28°36'	83°01'	3250	KY310779	KY310888	KY310939
kanjiroba **sp. nov.**	A10_12	Nepal, Baglung District	28°36'	83°01'	3250	KY310780	KY310889	KY310940
kanjiroba **sp. nov.**	A11_12	Nepal, Baglung District	28°36'	83°01'	3250	KY310781	KY310890	KY310941
kanjiroba **sp. nov.**	A12_12	Nepal, Baglung District	28°36'	83°01'	3250	KY310782	KY310891	KY310942
kanjiroba **sp. nov.**	A13_12	Nepal, Baglung District	28°36'	83°01'	3250	KY310783	KY310892	KY310943
kanjiroba **sp. nov.**	A14_12	Nepal, Baglung District	28°30'	83°02'	3073	KY310784	KY310893	KY310944
kanjiroba **sp. nov.**	A15_12	Nepal, Myagdi District	28°30'	83°07'	2993	KY310785	KY310894	KY310945
kanjiroba **sp. nov.**	A16_12	Nepal, Myagdi District	28°30'	83°07'	2993	KY310786	KY310895	KY310946
kanjiroba **sp. nov.**	A0570/99 [Scut3]*,#	Nepal, Talphi	29°18'	82°20'	2780	PP766265	–	–
kanjiroba **sp. nov.**	A0574/99 [Scut1]*	Nepal, Jumla District	29°06'	82°35'	3600	PP344607	PP359509	PP349711
kanjiroba **sp. nov.**	A0576/99 [Scut2]§	Nepal, Dolpa District	29°01'	82°47'	4450	KY310787	KY310896	KY310947
kanjiroba **sp. nov.**	A1248/05 [Scut5]*	Nepal, Jumla District	29°21'	82°23'	4450	PP344608	–	PP349712
kanjiroba **sp. nov.**	A1249/05 [Scut6]*	Nepal, Jumla District	29°21'	82°23'	4450	KY310788	KY310897	KY310948
kanjiroba **sp. nov.**	A1250/05 [Scut7]*	Nepal, Jumla District	29°21'	82°23'	4450	KY310789	–	KY310949
kanjiroba **sp. nov.**	A1251/05 [Scut8]*	Nepal, Jumla District	29°21'	82°23'	4450	KY310790	–	KY310950
kanjiroba **sp. nov.**	A1252/05 [Scut9]*	Nepal, Jumla District	29°21'	82°23'	4450	KY310791	KY310898	KY310951
kanjiroba **sp. nov.**	A1253/05 [Scut10]*	Nepal, Jumla District	29°23'	82°08'	2700	KY310792	–	KY310952
kanjiroba **sp. nov.**	A1254/05 [Scut11]*,#	Nepal, Jumla District	29°23'	82°08'	2700	PP344609	–	–
liupanensis	KIZNX080513	China, Ningxia, Guyuan, Jingyuan	36°33'	104°41'		–	JN700836	–
liupanensis	FWR11	China, Liupan Mountains	35°36'	106°7'		–	KC140483	JX533792
liupanensis	CPG8					–	KC140479	JX533802
luozhaensis	CIB_119122	China, Tibet, Luozha	28°23'	90°51'		OR469860	OR141848	–
luozhaensis	CJA20220121	China, Tibet, Luozha	28°23'	90°51'		OR469873	OR141849	–
luozhaensis	CIB_119115	China, Tibet, Luozha	28°23'	90°51'		OR469854	OR141832	–
mammatus	LC045	China, Sichuan, Baiyu	31°01'	99°15'	3494	KY310775	KY310884	KY310964
mammatus	XM949	China, Sichuan, Kangding, Jia Gen Ba	29°54'	101°35'	3350	–	MW021360	FJ463152
muliensis	KIZYPX36717					–	MW167046	MW133420
muliensis	KIZYPX36718					–	MW167047	MW133421
nepalensis	A2018/13	Nepal, Chainpur, Khaptad, Tribeni	29°22'	81°08'	3000	KY310777	KY310886	KY310937
nepalensis	A1724/09	Nepal, Chainpur, Khaptad, Tribeni	29°22'	81°08'	3000	PP766266	–	–
ningshanensis	HBY2	China, Shaanxi, Taibai, Huangbaiyuan	33°52'	107°31'	1652	–	KC140559	KC140561
ningshanensis	HBY3	China, Shaanxi, Taibai, Huangbaiyuan	33°52'	107°31'	1652	–	KC140560	KC140562
ningshanensis	nsc9	China, Shaanxi, Ningshan, Pingheliang	33°28'	108°31'	2000	–	KF757436	–
ningshanensis	srs17	China, Shaanxi, Lushan, Shirenshan	33°43'	112°15'	1642	–	KF757439	–
nyingchiensis	JS171001	Bhutan, Black Moutains	27°21'	90°20'	3940	PP344622	–	PP349725
nyingchiensis	JS171002	Bhutan, Black Moutains	27°21'	90°20'	3940	PP344623	–	PP349726
nyingchiensis	KIZ017459	China, Tibet, Nyingchi	29°39'	94°21'		–	KU243056	–
nyingchiensis	KIZ017460	China, Tibet, Nyingchi	29°39'	94°21'		–	KU243057	–
nyingchiensis	lz10	China, Tibet, Nyingchi, Bayi, Lulang	29°46'	94°44'		OM505486	OM506169	OM505283

nyingchiensis	BQ16	China, Tibet, Nyingchi, Bayi, Lulang	29°46'	94°44'		OM505493	OM506176	OM505290
nyingchiensis	BQ26	China, Tibet, Nyingchi, Bayi, Lulang	29°46'	94°44'		OM505496	OM506179	OM505293
occidentalis	Pk1	Pakistan, Deosai Plains	34°59'	75°15'	4100	PP344624	–	PP349727
occidentalis	Pk2	Pakistan, Deosai Plains	34°59'	75°15'	4100	KY310794	KY310899	KY310954
occidentalis	Pk5	Pakistan, Deosai Plains	34°59'	75°15'	4100	KY310795	KY310900	KY310956
occidentalis	Pk6	Pakistan, Deosai Plains	34°59'	75°15'	4100	KY310796	KY310901	KY310957
occidentalis	2009138	Pakistan, Lulusar	35°5'	73°55'		–	MZ073379	MZ126691
sikimmensis	KIZ011127	China, Tibet, Yadong	27°49'	89°4'		–	KU243058	MW133429
sikimmensis	KIZ07371	China, Tibet, Yadong	27°49'	89°4'		–	KU243059	MW133430
sikimmensis	KIZ013982	China, Tibet, Yadong	27°49'	89°4'		–	MW021367	MW133431
sikimmensis	KIZ013983	China, Tibet, Yadong	27°49'	89°4'		–	MW021368	MW133432
spinosus	KIZ011092					–	MW021362	MW133426
spinosus	KIZ011114	China, Tibet, Medog	29°42'	95°35'	2705	–	KU243053	MW133428
spinosus	KIZ011100	China, Tibet, Medog	29°42'	95°35'	2705	–	KU243054	MW13342
spinosus	KIZ012645	China, Tibet, Medog	29°42'	95°35'	2705	–	KU243055	–
tengchongensis	SYSa005799	China, Yunnan, Tengchong	25°1'	98°29'		MK121789	MK121783	–
tengchongensis	SYSa005800	China, Yunnan, Tengchong	25°1'	98°29'		MK121790	MK121784	–
tengchongensis	SYSa005801	China, Yunnan, Tengchong	25°1'	98°29'		MK121791	MK121785	–
tuberculatus	CIBXM988	China, Sichuan, Yuexi, Puxiong	28°31'	102°45'	2900	EF397278	–	FJ945493
tuberculatus	XM995	China, Sichuan,Yuexi, LaJi	28°5'	102°49'	2520	–	MW021351	FJ945492
tuberculatus	KIZYPX11046					–	MW021397	MW133413
wanglangensis	21514_1	China, Sichuan, Mianyang	31°27'	104°43'		–	OQ361635	–
wanglangensis	21514_2	China, Sichuan, Mianyang	31°27'	104°43'		–	OQ361636	–
wanglangensis	21505_4	China, Sichuan, Mianyang	31°27'	104°43'		–	OQ361637	–
wuguanfui	KIZ011101	China, Tibet, Medog	29°42'	95°35'	2705	–	KU243060	MW133433
wuguanfui	KIZ011102	China, Tibet, Medog	29°42'	95°35'		–	KU243061	MW133434
Oreolalax chuanbeiensis	DQR-Pingwu-001J					EU180887	–	EU180929
O. omeimontis	KIZ-O.O96002					EU180886	–	EU180928
O. xiangchengensis	CIB20130642					MH727696	MH727696	MH727696

Table S3. List of samples used in this study for phylogenetic network analysis. Accession numbers of sequences generated in this study are indicated by grey cells. §Holotype: *S. kanjiroba* **sp. nov.** A0576/99 (lab ID Scut2); *paratype.

species	*ID*	*Locality*	*Lat*	*Long*	*Alt*	*bfib7*	*ccnb2*	*rag1*
boulengeri	KQ16_14	China, Sichuan	30°22'	101°40'	4062	KY310817	–	KY311035
boulengeri	A1_AL	China, Tibet	29°36'	85°44'	5067	KY310818	KY352195	KY311036
boulengeri	A2_AL	China, Tibet	29°36'	85°44'	5067	KY310819	KY352196	KY311037
boulengeri	A3_AL	China, Tibet	30°09'	90°38'	4887	KY310820	–	KY311038
boulengeri	A5_AL	China, Tibet	30°09'	90°38'	4887	KY310821	–	KY311039
chintingensis	LC141 01_6/01_9	China, Sichuan, Tianquan	30°14'	102°30'	2500	KY310824-25	KY352197-98	KY311042-43
chintingensis	JF174	China, Sichuan	29°39'	102°57'		FJ945585	FJ945677	–
chintingensis	ROM40460	China, Sichuan	29°39'	102°57'		–	–	EF397301
chintingensis	ROM39065	China, Sichuan, Hongya	29° 54'	103° 22'		–	–	EF397303
glandulatus	2014_Sc1	China, Sichuan, Garzê	31°40'	99°43'	3457	KY310826	KY352199	KY311044
glandulatus	2014_Sc2	China, Sichuan, Garzê	29°47'	100°25'	3713	KY310827	KY352200	KY311045
glandulatus	SH150557	China, Sichuan, Garzê	29°47'	100°25'	3713	KY310831	–	KY311049
glandulatus	CIB_ZYCB736	China, Sichuan, Yajiageng	29° 54'	102°0'		MW194104	–	–
glandulatus	2014_KQ61	China, Sichuan, Garzê	29°47'	100°25'	3713	KY310828	KY352201	KY311046
glandulatus	XM1188	China, Sichuan, Shagong	29°09'	99°56'	4000	–	FJ945646	EF397298
glandulatus	IOZ4198	China, Sichuan, Sangzui	29°11'	100°06	4000	FJ945554	FJ945645	–
glandulatus	XM1190					–	FJ945644	–
glandulatus	XM1191					–	FJ945643	–
glandulatus	IOZ4240	China, Sichuan, Changhaizi	29°54'	101°60'	3815	FJ945558	FJ945642	–
glandulatus	SH150516	China, Sichuan, Tagong	30°14'	101°30'	3526	KY310829	–	KY311047
glandulatus	SH150531	China, Sichuan, Xinduqiao	30°02'	101°32'	3474	KY310830	–	KY311048
gongshanensis	CAS234295					–	–	KX208788
kanjiroba **sp. nov.**	A2014/13*	Nepal, Jumla District	29°21'	82°09'	3285	KY310833	–	KY311051
kanjiroba **sp. nov.**	A8_12	Nepal, Rukum District	28°42'	82°55'	3340	KY310835	KY352204	KY311053
kanjiroba **sp. nov.**	A9_12	Nepal, Baglung District	28°36'	83°01'	3250	KY310836	KY352205	KY311054
kanjiroba **sp. nov.**	A10_12	Nepal, Baglung District	28°36'	83°01'	3250	KY310837	–	KY311055
kanjiroba **sp. nov.**	A11_12	Nepal, Baglung District	28°36'	83°01'	3250	–	KY352206	KY311053
kanjiroba **sp. nov.**	A12_12	Nepal, Baglung District	28°36'	83°01'	3250	KY310838	KY352207	KY311056
kanjiroba **sp. nov.**	A13_12	Nepal, Baglung District	28°36'	83°01'	3250	KY310839	KY352208	KY311057
kanjiroba **sp. nov.**	A14_12	Nepal, Baglung District	28°30'	83°02'	3073	KY310840	KY352209	KY311058
kanjiroba **sp. nov.**	A15_12	Nepal, Myagdi District	28°30'	83°07'	2993	KY310841	KY352210	KY311059
kanjiroba **sp. nov.**	A0574/99* Scut1*	Nepal, Jumla District	29°06'	82°35'	3600	PP349666	–	–
kanjiroba **sp. nov.**	A0576/99 Scut2§	Nepal, Dolpa District	29°01'	82°47'	4450	–	–	KY311051
kanjiroba **sp. nov.**	A1248/05 Scut5*	Nepal, Jumla District	29°21'	82°23'	4449	KY310842	KY352211	KY311060
kanjiroba **sp. nov.**	A1249/05 Scut6*	Nepal, Jumla District	29°21'	82°23'	4450	KY310843	–	KY311061
kanjiroba **sp. nov.**	A1250/05 Scut7*	Nepal, Jumla District	29°21'	82°23'	4450	KY310844	–	KY311062
kanjiroba **sp. nov.**	A1251/05 Scut8*	Nepal, Jumla District	29°21'	82°23'	4450	KY310845	KY352212	KY311063
kanjiroba **sp. nov.**	A1252/05 Scut9*	Nepal, Jumla District	29°21'	82°23'	4450	–	KY352213	KY311064
kanjiroba **sp. nov.**	A1253/05 Scut10*	Nepal, Jumla District	29°23'	82°08'	2700	PP349667		PP349689
kanjiroba **sp. nov.**	A1254/05 Scut11*	Nepal, Jumla District	29°23'	82°08'	2700	PP349668		
liupanensis	KIZNX080519					–	–	MW111376
luozhaensis	CIB_119115	China, Tibet, Luozha	28°14'	90°47'	4150	–	–	OR546320
luozhaensis	CIB_119116	China, Tibet, Luozha	28°14'	90°47'	4150	–	–	OR546321
luozhaensis	CIB_119117	China, Tibet, Gari, Se Town	28°13'	90°49'	3970	–	–	OR546322
mammatus	LC045	China, Sichuan, Baiyu	31°01'	99°15'	3494	KY310832	KY352202	KY311050
mammatus	LC066	China, Sichuan, Bai Yu	31°01'	99°15'	3494	FJ945583	FJ945674	–
mammatus	XM949	China, Sichuan, Jia Gen Ba	29°54'	101°35'	3350	FJ945582	FJ945676	–
mammatus	XM972	China, Sichuan, Xinduqiao	30°01'	101°28'	3350	FJ945581	FJ945675	EF397300
muliensis	IOZCAS3638	China, Sichuan, Bailingshan	27°20'	101°32'	3866	–	–	EF397302
nepalensis	A2018/13	Nepal, Chainpur, Khaptad	29°22'	81°08'	3000	KY310834	KY352203	KY311052
nepalensis	A1724/09	Nepal, Chainpur, Khaptad	29°22'	81°08'	3000	PP755129	PP755130	PP755131
nyingchiensis	KIZ017459	China, Tibet, Nyingchi	29° 39'	94° 21'		–	–	MW111377
occidentalis	Pk1	Pakistan, Deosai Plains	34°59'	75°15'	4100	KY310846	KY352214	KY311065
occidentalis	Pk2	Pakistan, Deosai Plains	34°59'	75°15'	4100	KY310846	KY352215	PP349708
occidentalis	Pk4	Pakistan, Deosai Plains	34°59'	75°15'	4100	KY310847	KY352216	KY311066
occidentalis	Pk5	Pakistan, Deosai Plains	34°59'	75°15'	4100	KY310846	KY352217	PP349709
occidentalis	Pk6	Pakistan, Deosai Plains	34°59'	75°15'	4100	KY310847	KY352218	KY311066
occidentalis	ZFMK103262	Pakistan, Astore	35°14'	74°43'	2941	–	–	MZ126688
occidentalis	SH2009138	Pakistan, Lulusar	35°04'	73°55'	3399	–	–	MZ126689
tuberculatus	KIZYPX11046					–	–	MW111379
tuberculatus	IOZ4187	China, Sichuan, Yading	28°26'	100°21'	3997	–	FJ945652	–
tuberculatus	XM988	China, Sichuan, Puxiong	28°31'	102°45'	2900	FJ945565	FJ945651	–
tuberculatus	XM989	China, Sichuan, Puxiong	28°31'	102°45'	2900	FJ945564	FJ945650	–
tuberculatus	XM995	China, Sichuan, LaJi	28°5'	102°49'	2520	FJ945563	FJ945649	–
tuberculatus	IOZ3554	China, Sichuan, Yele	28°55'	102°11'	2691	–	FJ945648	–
tuberculatus	IOZ3555	China, Sichuan, Yele	28°55'	102°11'	2691	FJ945566	FJ945647	–
wuguanfui	KIZ011102	China, Tibet, Medog	29°42'	95°35'	2705	–	–	MW111378

Table S4. Uncorrected p-distance (upper diagonal) and maximum composite likelihood distance values (lower diagonal) for COI sequences from phylogenetic analysis of species of the genus *Scutiger* (Anura: Megophryidae). Distances ≤3.5% are indicated by grey-shaded cells. The newly described species is indicated bold.

	Scutiger	1	2	3	4	5	6	7	8	9	10	11	12	13	14	15	16	17	18	19	20	21	22	23
1	*feiliangi*		12.4	12.3	13.4	12.1	13.3	12.7	13.8	13.4	13.7	13.4	13.1	12.4	13.4	**13.2**	13.8	3.0	12.5	12.7	12.9	11.7	13.3	13.8
2	*tuberculatus*	10.4		5.3	12.2	7.2	13.2	12.9	13.2	8.2	8.1	13.5	8.0	7.8	8.4	**13.0**	12.1	13.1	13.2	10.2	13.5	11.8	15.0	13.5
3	*muliensis*	10.3	4.3		12.1	7.1	14.3	13.1	13.2	8.2	7.9	14.7	7.1	7.5	8.7	**12.1**	12.1	13.7	13.6	10.8	14.2	11.9	15.9	12.3
4	*chintingensis*	11.3	11.0	10.7		12.2	13.1	12.8	11.5	12.6	12.6	13.7	12.2	11.3	12.7	**12.5**	12.6	13.0	12.7	11.6	13.1	11.6	14.9	13.2
5	*wanglangensis*	10.3	6.0	5.4	11.4		12.3	12.8	11.8	6.5	7.4	13.6	4.8	2.4	8.0	**11.1**	10.8	12.5	12.5	10.6	13.2	11.4	14.6	12.7
6	*tengchongensis*	10.8	10.7	11.7	11.2	10.7		10.3	13.1	12.3	12.5	11.1	12.7	10.5	12.3	**12.1**	12.8	13.8	10.6	12.2	13.3	9.2	15.8	10.7
7	*luozhaensis*	10.5	12.3	11.9	11.8	12.4	9.3		11.5	13.4	12.9	7.8	12.7	12.5	13.8	**11.5**	12.6	12.6	6.9	12.7	12.3	9.9	15.6	11.2
8	*ghunsa*	11.9	12.1	11.5	10.3	11.0	10.5	10.2		12.2	11.8	11.5	12.3	11.4	12.0	**9.2**	9.4	14.1	11.5	10.8	12.7	10.2	14.8	12.5
9	*boulengeri*	11.4	6.9	6.1	11.6	5.4	10.2	12.7	10.4		7.3	13.9	5.9	6.2	3.4	**12.6**	11.4	13.7	12.5	10.8	14.1	12.2	15.0	11.8
10	*glandulatus*	11.6	7.1	6.7	12.1	6.6	10.4	12.4	10.9	6.0		14.2	6.7	7.3	7.7	**11.7**	12.3	14.5	13.0	11.1	13.4	11.5	16.0	11.9
11	*gongshanensis*	11.6	12.1	12.6	12.1	12.9	9.6	6.7	9.9	12.9	13.5		14.0	13.6	13.9	**12.9**	13.1	14.0	8.1	13.3	12.4	10.1	16.7	12.4
12	*jiulongensis*	11.1	7.4	5.9	11.3	4.5	11.0	12.1	11.5	4.6	5.9	12.8		4.5	7.8	**12.6**	11.2	13.3	12.7	10.0	14.0	12.5	16.0	11.4
13	*liupanensis*	10.2	6.6	5.9	10.1	1.9	9.3	11.8	9.7	4.7	5.7	12.1	4.0		8.0	**11.7**	10.6	12.8	12.1	10.2	12.7	10.9	14.1	11.6
14	*mammatus*	11.3	7.2	6.3	11.8	6.7	10.4	13.2	10.1	2.6	6.6	13.1	6.2	6.1		**12.2**	11.6	13.8	12.6	10.7	14.1	12.4	14.6	11.5
**15**	***kanjiroba* sp. nov.**	**10.7**	**11.5**	**10.3**	**10.9**	**10.3**	**10.1**	**11.7**	**7.8**	**11.2**	**11.0**	**12.0**	**11.6**	**10.0**	**10.9**		**7.4**	**14.1**	**11.7**	**11.9**	**13.1**	**9.4**	**15.5**	**11.0**
16	*nepalensis*	11.7	10.6	9.6	10.9	9.4	10.1	11.8	8.0	9.5	10.3	12.6	9.3	8.3	9.7	**5.9**		15.2	12.0	11.1	14.6	10.5	14.6	11.9
17	*ningshanensis*	2.0	11.1	11.1	11.5	11.2	11.3	10.7	11.9	12.4	12.5	11.8	11.8	11.0	12.2	**11.7**	12.8		13.3	13.3	12.8	12.5	13.8	14.3
18	*nyingchiensis*	10.1	12.0	11.8	11.2	11.3	8.7	6.3	9.5	10.9	11.4	7.3	11.4	10.4	11.0	**11.1**	11.1	10.7		12.2	12.4	9.5	14.3	10.6
19	*occidentalis*	10.6	9.1	8.8	10.5	10.1	10.3	10.8	8.5	10.1	9.9	11.8	9.3	9.5	10.2	**9.5**	8.8	11.2	10.3		12.4	11.3	15.6	11.2
20	*sikimmensis*	10.9	11.8	12.3	12.0	12.5	12.2	11.6	11.0	13.1	12.7	12.0	13.0	11.8	13.2	**12.3**	13.1	10.9	11.4	11.1		12.0	14.3	11.1
21	*spinosus*	9.9	10.3	10.2	10.0	10.5	7.8	8.7	8.2	10.7	10.2	8.6	11.7	9.9	10.8	**7.3**	8.6	10.5	7.7	10.2	11.0		14.6	11.2
22	*wuguanfui*	11.5	12.0	12.8	12.4	12.4	12.7	14.2	12.5	12.5	13.4	14.4	13.7	11.9	12.2	**13.5**	11.7	12.3	12.1	12.7	12.3	12.0		15.0
23	*bhutanensis*	11.6	11.8	10.2	12.0	11.7	9.9	10.7	10.8	10.6	10.6	11.6	9.9	10.1	10.8	**9.6**	9.9	12.1	9.7	10.0	9.8	10.3	12.2	

Table S5. Morphometric, meristic, and categorical data for adults and labial tooth row formula of tadpoles of *Scutiger kanjiroba* **sp. nov.** compared with congeneric species; all measurements in mm. SVL: Snout–vent length; HL: head length; HW: head width; SL: snout length; IND: internarial distance; HAL: hand length; 1: relative length of fingers; 2: relative length of toes; 3: subarticular tubercles present (1)/absent (0); 4: vocal sac present (1)/absent (0); 5: vomerine/maxillary dentation present (1) or absent (0); 6: tympanum present (1)/absent (0); 7: webbing toes (rud.=rudimentary); 8: finger(s) with nuptial spines (males); 9: pectoral/axillary glands (males); 10: gland size (p, ax); 11: spines on p/ax present (1) or absent (0); 12: spines on (inner) (fore)arms present (1)/absent (0); 13: belly with (1) or without (0) spines; 14: tubercles/warts on dorsal (and lateral) surfaces of body and/or limbs present (1) or absent (0); 15: dorsal tubercles with (1) or without (0) black spines in breeding condition (males).*in male *S. kanjiroba* sp. nov.: spines on p/ax light, probably faded due to preservation.

data source (the 1st represents the original description)	Scutiger	SVL♂	SVL♀	HL	HW	SL	IND	HAL	1	2	3	4	5	6	7	8	9	10	11	12	13	14	15	LTRF tadpole	alt
This study	*kanjiroba* **sp. nov.**	55.4 (n=1)	54.2-66.7 (n=3)	14.5-19.1	18.2-23.2	7.9-9.6	5.6-6.8	13.0-16.5	I<II<IV<III	I<II<V<III<IV	0	0	0/0	0	rudimentary	I,II,III	2/2	p≈ax	1/1*	0	0	1	1	1:2+2/2+2:1	3000-4400
(Dubois, 1979)	*adungensis*	71.0-73.0 (n=2)			24-24.5		5.0-6.0		I=II<IV<III		0	1	0/1	0	rudimentary	I,II	2/0		1/0		0	0	0		3650
(Rao, 2022 "2020")	*bangdaensis*	45.5-50.0 (n=2)	48-49.5 (n=2)											0	developed	I,II,III	2/2	p>ax	1/1		0	1	0		
(Delorme and Dubois, 2001)	*bhutanensis*	53.0-53.4 (n=2)		18.1	18.9	12.8	4.6	12.8	I=II<IV<III	I<II<III=V<IV	0 or 1	0	0/0	0	0, rud., or weak	I,II	2/2	p=ax	1/1		*na*	1	1		
(Rao, 2022 "2020")	*biluoensis*	73.0 (n=1)	53.5 (n=1), sa										1	0	rudimentary	I,II	2/2		1/1	1	0				3800
(Bedriaga, 1898); data partly from (Fei et al., 2009; Fei et al., 2012)	*boulengeri*	44.9-53.7 (n=20)	40.2-58.2 (n=8)						I=II<IV<III			0	0/0 or 1	0	developed	I,II,III	2/2	p=ax	1/1		1	1	1	1:6+6/6+6:1	3850
(Liu, 1950)	*brevipes*	68.0-80.0 (n=10)	58.0-68.0 (n=9)	23.0	24.0				I=II<IV<III		0 (indistinct)	0	0/0		developed	I,II	2/2	p>>ax	0/0		0	1		1:4+4/5+5:1	3500
Liu and Hu, 1960; data partly from (Fei et al., 2009)	*chintingensis*	42.0-42.4 (n=3)	48.0-52.8 (n=6)	13.6-16.0	14.2-16.0	5.0-6.0	4.0-4.8	11.0-13.0	I=II<IV<III		0 (indistinct)	0	0/1	0	developed (large or reduced)	I,II,III	2/2	p>ax	1	1	0	1		1:3+3/2+2:1	3050
(Zhou et al., 2023)	*feiliangi*	45.7-50.2 (n=6)	48.9-51.5 (n=3)	13.6-17.6	15.3-20.0	5.8-6.8	3.3-4.1		I<II<IV<III	I<II<V<III<IV	0	0	*na*/1	"indistinct"	rudimentary	I,II,III	2/2 (♂♀)	p>ax	1/1 (♂♀)	1	0 (1 in ♀)	1	1	1:5+5/5+5:1	1882
(Khatiwada et al., 2019)	*ghunsa*	42.0–47.8 (n=5)	50.2 –53.9 (n=5)	10.1-13.3	14.4-18.0	4.7-6.2	3.5-4.9	19.9-25.1	I<II<IV<III	I<II<III<V<IV	0	0	0/0	1 (hidden)	0	I,II,III	2/2	p>>ax	1/1		0	1	1	1:2+2 /2+2:1	3457
(Liu, 1950)	*glandulatus*	67.0-81.0 (n=2)	77.0-81.0 (n=2)	28.0-26.0	28.5-28.0				I=II<IV<III	I<II<III=V<IV	1	0	0/0	0	developed	I,II	2/2	p>>ax	1/0		0	1		1:(3-4)+(3-4)/(4-5)+(4-5):1	2290-2740
(Yang et al., 1979); data from (Jiang et al., 2016)	*gongshanensis*	47.0-57.0 (n=19)	49.0-60.5 (n=2)						I=II<IV<III		0	1	0/1	0	0	I,II	2/0		1		0	1	0	1:3+3/3+3:1	2750
(Fei et al., 1995); data from (Fei et al., 2012; Jiang et al., 2016)	*jiulongensis*	67.4-81.5 (n=20)									1	0	*na*/0	0	weak	I,II	2/2	p>>ax	1/1		0	1		1:3+3/4+4(3+3):1	3210
(Huang, 1985)	*liupanensis*	40.6-48.0 (n=20)	52.0-59.5 (n=3)	12.8-16.3	13.6-17.8	5.1-6.5	3.5-5.0	11.0-15.9	I=II<IV<III	I<II<III=V<IV		0	*na*/1	0	developed	I,II,III	2/2	p=ax	1/1		1	1	1	1:6+6 (5+5)/6+6 (5+5) (7+7):1	2100
(Shi et al., 2023)	*luozhaensis*	47.0–57.9 (n=19)	49.8-66.2 (n=8)	15.4-18.8	16.4-23.3	6.3-8.2	4.2-6.3	11.6-16.8				0	0/0 (or indistinct)	0	rudimentary	I,II,III	2/2	p>ax	1/1	0	0	1	1	1:3+3/3+3 (2+2):1	4150
(Liu, 1950); data partly from (Fei et al., 2009)	*maculatus*	49 (n=1)		16.0	17.0			13.0	I<II<IV<III		1	0	0/0 or 1	0	developed	I,II,III	2/2	p>ax	1/1		0	1			3353
(Günther, 1896); data from (Dubois, 1979; Liu, 1950)	*mammatus*	58.8-71.7 (n=11)	63.0-77.3 (n=6)						I=II<IV<III		1 (or very indistinct)	0	0/0 or 1	0	developed (large or reduced)	I,II	2/2 or 2/0	p>ax or p	1/0 or 0/0		0			1:5+5/5+5:1	
(Rao, 2022 "2020")	*meiliensis*	70.0 (n=1)	65.0 (n=1)										1	0	rudimentary	I,II	2/2		1/1	0	0				3800
(Fei and Ye, 1986); data partly from (Fei et al., 2009)	*muliensis*	68.2-80.0 (n=10)	60.1-67.5 (n=10)						I=II<IV<III	I<II<III=V<IV		0	0/0	0	rudimentary	I,II	2	p or ax?	1		0			1:4+4/4+4 (5+5):1	3200
(Dubois, 1974 "1973") (Dubois, 1978)	*nepalensis*	68.0-73.5 (n=4)	59.5-74.5 (n=4)						I=II<IV<III	I<II<V<III<IV	0	0	na	0	0 or rudimentary	I,II,III	2/2	p=ax or p>ax	1/1		0	1	1		
(Fang, 1985); data partly from (Fei et al., 2012; Jiang et al., 2016)	*ningshanensis*		41.0 (n=1)	14.0	15.0	5.0	3.2					0	na/1	"not visible"	weak	I,II,III	2/2	p=ax			1			1:5+5/5+5:1	2550
(Fei, 1977); data partly from (Fei et al., 2009)	*nyingchiensis*	52.8-67.6 (n=5)	70.0 (n=1)						I=II<IV<III			0	0/1	0	developed	I,II,III	2/2	p>ax	1/1		0	1	1	1:4+4 (3+3)/4+4:1	3040
(Dubois, 1978)	*occidentalis*	52.0-71.5 (n=63)	58.5-73.0 (n=12)	23.5	24.5				I<II<IV<III	I<II<III<V<IV	0		0/0	0	developed	I,II,III	2/2	p>ax	1/1	1	0	1	1 (also breeding ♀)	1:4+4 (3+3)/4+4 (3+3):1	2680-3900
(Liu et al., 1978); data partly from (Fei et al., 2012; Jiang et al., 2016)	*pingwuensis*	60.7-75.8 (n=20)									0	0	na/0	0	weak	I,II,III	2/2	p>>ax		1	1			1:2+2/3+3:1	2200
(Blyth, 1855 "1854"; Boulenger, 1987); data partly from (Fei et al., 2012) and (Che et al., 2020) [*S. sikimmensis* from Yadong]	*sikimmensis*	46.9-51.3 (n=8)	50.8-53.9 (n=2)	16.0-17.5	16.7-19.0	5.7-6.3	4.0-4.8	11.6-14.3	I=II<IV<III	I<II<III<V<IV	0	0	0/0 or 1	0	0, rudimentary, or weak; well-developed in Che et al. 2020	I,II,III	2/2	p>ax	1/1		0	1	0 (or 1: Fei et al. 2009)	1:2+2 (or 3+3)/3+3:1	3350
(Das and Chanda, 2000): ZSI9854, p. 71		54.4 (n=1)									0				rudimentary		2/2					1			
(Jiang et al., 2016)	*spinosus*	50.5-55.6 (n=12)	53.8-57.2 (n=4)	17.4-19.8	17.3-19.8	3.9-7.9	4.0-4.8	13.2-16.1	I=II<IV<III	I<II<V<III<IV	0	0	0/0 or indistinct	0	rudimentary	I,II,III	2/2	p>>ax	1/1	1	0	1	1 (♂♀)		2705
(Yang and Huang, 2019)	*tengchongensis*	36.0–40.1 (n=7)		12.0-14.0	11.5-13.8	5.1-5.3	3.2-3.4	8.0-9.4	I=II<IV<III	I<II<III=V<IV	0 (indistinct)	0	0/0	0	rudimentary	I,II,III	2/2	p>>ax	1/1	0	0	1	1	1:1+2 (2+2)/3+):1	3000
(Liu and Fei, 1979); data partly from (Fei et al., 2009)	*tuberculatus*	68.0-76.0 (n=16)	63.6-79.0 (n=7)						I=II<IV<III		0		na/0	0	rudimentary	I,II	2/2	p>>ax			0	1		1:2+2(or 3+3)/3+3:1	3000
(Ye and Fei, 2007); data partly from (Fei et al., 2009)	*wanglangensis*	52.7-58.2 (n=6)	64.3 (n=1)						I=II<IV<III		0		0/1	0	rudimentary or weak	I,II,III	2/2	p>ax			1				2500
(Jiang et al., 2012)	*wuguanfui*	77.5−83.8 (n=5)	116.7 (n=1)	28.8-40.3	29.6-47.6	6.0-10.3	6.5-10.1	20-27.8	I=II<IV<III	I<II<III=V<IV	0 (indistinct)	1	0/0	0	weak	I,II,III	2/2	p=ax	1	0	0	1	1	I:3+3/3+3:1	2705

Table S6. Morphological evaluation of *S. nepalensis*, *S. kanjiroba* **sp. nov.,** and *S. ghunsa* (for *S. ghunsa*: data from literature). 1: relative length of fingers; SVL: Snout–vent length, for *S. nepalensis* the values from the original description are given in brackets; HL: head length; HW: head width; SL: snout length; NSD: distance of naris (center) to tip of snout; ED: horizontal diameter of eye; END: eyelid-naris distance; IND: internarial distance; FAL: forearm length; HAL: hand length; FEL: femur (thigh) length; TIBL: tibia (shank) length; TaL: length of tarsus; FOL: foot length; 1: relative length of fingers; 2: relative length of toes; 3: subarticular tubercles present (1)/absent (0); 4: vocal sac present (1)/absent (0); 5: vomerine/maxillary dentation present (1) or absent (0); 6: tympanum present (1)/absent (0); 7: webbing toes (rud.=rudimentary; 0=absent); 8: finger(s) with nuptial spines (males); 9: pectoral/axillar glands (males); 10: gland size (p, ax); 11: spines on p/ax present (1) or absent (0); 12: spines on (inner) (fore)arms of males present (1)/absent (0); 13: belly with (1) or without (0) spines; 14: tubercles/warts on dorsal (and lateral) surfaces of body and/or limbs present (1) or absent (0); 15: dorsal tubercles with (1) or without (0) black spines in breeding condition (males). Age: a=adult, sa=subadult, j=juvenil, mm=young metamorphling, tp=tadpole; sex: m=male, f=female.

source	ID	species	type	N	E	age	sex	SVL	HL	HW	HW/HL	SL	NSD	ED	ED/HL	END	IND	FAL	HAL	FEL	TIBL	TaL	FOL	1	2	3	4	5	6	7	8	9	10	11	12	13	14	15
MNHN Paris	1974.1096	*nepalensis*	para	29.30	80.99	a	m	67.45	21.87	26.13	1.19	9.27	4.63	6.29	0.29	4.57	6.57	16.64	17.56	28.16	27.31	16.65	29.70	3>4>2=1	4>3>5>2>1	0	0	0/0	0	rud.	I,II,III	2/2	p>ax	1	0	0	1	1
MNHN Paris	1974.1097	*nepalensis*	para	29.30	80.99	a	f	64.27 [64.50]	21.89	26.88	1.23	9.16	4.05	7.02	0.32	4.34	6.37	15.68	17.67	26.03	24.89	15.54	30.00	3>4>2>1	4>3>2>5>1	0	0	0/0	0	rud.	0	0	—	—	0	0	1	—
MNHN Paris	1974.1098	*nepalensis*	para	29.30	80.99	a	f	58.00 [59.50]	19.46	24.48	1.26	7.20	3.44	5.99	0.31	4.07	5.80	14.23	15.65	23.93	23.43	14.10	26.62	3>4>2>1	4>3>2>5>1	0	0	0/0	0	rud.	0	0	—	—	0	0	1	—
MNHN Paris	1989.3361	*nepalensis*	para	29.30	80.99	a	m	69.51	20.54	27.33	1.33	9.35	3.78	6.58	0.32	4.84	6.48	18.97	18.95	27.75	29.22	17.64	31.00	3>4>2>1	4>3>5>2>1	0	0	0/0	0	rud.	I,II,III	2/2	p>ax	1	0	0	1	(1;a few)
MNHN Paris	1989.3362	*nepalensis*	para	29.30	80.99	a	m	72.09 [73.50]	22.80	27.71	1.22	9.66	5.11	7.28	0.32	4.70	6.80	19.14	20.24	28.33	31.84	19.68	33.70	3>4>2>1	4>3>5>2>1	0	0	0/0	0	rud.	I,II,III	2/2	p>ax	1	0	0	1	1
NHME	A1724/09	*nepalensis*		29.37	81.14	a	f	57.66	19.10	21.31	1.11	8.32	4.75	5.67	0.30	3.95	5.62	16.05	12.71	21.26	19.5	14.12	22.75	3>4>2>1	4>3>5>2>1	0	0	0/0	0	rud.	0	0	—	—	0	0	1	0
NHME	A2018/13	*nepalensis*		29.37	81.14	j																																
NHME	A0576/99	*kanjiroba* **sp. nov.**	holo	29.02	82.79	a	m	55.40	15.37	19.14	1.25	7.88	3.44	6.00	0.39	4.00	5.75	13.60	13.04	23.00	21.16	13.34	23.53	3>4>2>1	4>3>5>2>1	0	0	0/0	0	0	I,II,III	2/2	p>/=ax	1/1	0	0	1	0-1
NHME	A0570/99	*kanjiroba* **sp. nov.**	para	29.30	82.34	a	f	66.20	19.10	23.20	1.22	9.60	5.70	6.40	0.34	4.30	6.40	14.60	16.40	25.90	23.40	15.00	27.60	3>4>2>1	4>3>5>2>1	0	0	0/0	0	0	—	0	—	—	0	0	1	0
NHME	A0574/99	*kanjiroba* **sp. nov.**	para	29.10	82.58	a	f	66.71	18.05	22.01	1.22	9.62	5.53	6.52	0.36	3.87	6.76	15.50	16.53	26.36	22.31	17.00	29.00	3>4>2>1	4>3>5>2>1	0	0	0/0	0	0	—	0	—	—	0	0	1	1
NHME	A1248/05	*kanjiroba* **sp. nov.**	para	29.36	82.40	a	f	54.16	14.53	18.22	1.25	8.06	5.33	5.0	0.34	3.42	5.59	11.82	13.96	21.54	20.27	12.07	23.00	3>4>2>1	4>3>5>2>1	0	0	0/0	0	0	—	0	—	—	0	0	1	0
NHME	A1249/05	*kanjiroba* **sp. nov.**	para	29.36	82.40	sa		43.76	13.53	13.78	1.02	6.82	3.16	4.27	0.32	3.10	4.82	10.17	10.85	17.37	16.65	10.59	17.84	3>4>2>1	4>3>5>2>1	0	0	0/0	0	0	—	0	—	—	0	0	1	0
NHME	A1250/05	*kanjiroba* **sp. nov.**	para	29.36	82.40	tp																																
NHME	A2014/13	*kanjiroba* **sp. nov.**	para	29.35	82.17	mm																																
NHME	A1247/05	*kanjiroba* **sp. nov.**	para	29.30	82.34	j																																
NHME	A1251/05	*kanjiroba* **sp. nov.**	para	29.36	82.40	j																																
NHME	A1252/05	*kanjiroba* **sp. nov.**	para	29.36	82.40	j																																
NHME	A1253/05	*kanjiroba* **sp. nov.**	para	29.39	82.15	j																																
NHME	A1254/05	*kanjiroba* **sp. nov.**	para	29.34	82.39	j																																
Khatiwada et al. 2019	2015-118	*ghunsa*	holo	27.66	87.93	a	m	43.60	11.40	16.40	1.44	4.90	*na*	4.80	0.42	3.10	4.10	10.00	23.40	17.50	17.10	11.10	19.30	3>4>2>1	4>3>5>2>1													
Khatiwada et al. 2019	2015-119	*ghunsa*	para	27.66	87.93	a	m	47.80	11.40	15.20	1.33	4.90	*na*	4.50	0.39	3.70	3.70	11.50	21.80	19.40	17.00	13.10	21.40	3>4>1>2	4>5>3>2>1													
Khatiwada et al. 2019	2015-120	*ghunsa*	para	27.66	87.93	a	m	45.60	10.10	15.40	1.52	4.70	*na*	5.60	0.55	2.30	3.50	9.40	21.40	19.40	17.60	11.40	20.10	3>2>1>4	4>3>5>2>1													
Khatiwada et al. 2019	2015-121	*ghunsa*	para	27.66	87.93	a	m	47.30	11.70	14.90	1.27	4.70	*na*	6.00	0.51	2.40	3.50	12.10	19.90	16.50	13.60	8.10	18.90	3>4>2>1	4>3>5>2>1													
Khatiwada et al. 2019	2015-122	*ghunsa*	para	27.66	87.93	a	m	42.10	10.40	14.40	1.38	4.70	*na*	4.80	0.46	2.70	3.60	8.90	20.30	13.40	16.00	10.40	18.60	3>4>2>1	4>5>3>2>1													
Khatiwada et al. 2019	2015-123	*ghunsa*	para	27.66	87.93	a	f	53.60	13.30	17.80	1.34	6.20	*na*	7.20	0.54	3.20	4.00	10.10	25.10	20.60	17.40	13.10	22.40	3>4>2>1	4>3>5>2>1													
Khatiwada et al. 2019	2015-124	*ghunsa*	para	27.66	87.93	a	f	50.20	13.20	15.50	1.17	6.10	*na*	6.20	0.47	3.40	4.90	11.90	21.60	16.30	15.30	12.30	22.20	3>4>2=1	4>3>5=2>1													
Khatiwada et al. 2019	2015-125	*ghunsa*	para	27.66	87.93	a	f	53.90	12.80	18.00	1.41	6.20	*na*	5.00	0.39	3.50	4.60	12.00	23.70	20.00	18.70	7.80	22.80	3>4>2>1	4>5>3>2>1													

Table S7. TukeyHSD post hoc tests for significant ANOVA results ($p<0.05$) on the morphometric characters (normalized by SVL) of *Scutiger ghunsa*, *S. kanjiroba* **sp. nov,** and *S. nepalensis*; P-values <0.05 are indicated bold. SVL: Snout–vent length; SL: snout length; HW: head width; HL: head length; IND: internarial distance; FAL: forearm length; HAL: hand length; TIBL: tibia (shank) length; FOL: foot length. Diff: difference in the observed means; lwr: lower end point of the interval; upr: upper end point; p adj: p-value after adjustment for the multiple comparisons. Significance codes: 0 '***'; 0.001 '**'; 0.01 '*'; 0.05 '.'

ANOVA Pr(>F)		diff	lwr	upr	P adj
SVL: 7.33e-05***	*kanjiroba-ghunsa*	12.605	4.180	21.030	**0.004**
	nepalensis-ghunsa	18.252	10.408	26.095	**0.000**
	nepalensis-kanjiroba	5.647	-3.582	14.875	0.278
SL: 1.92e-05***	*kanjiroba-ghunsa*	0.035	0.022	0.048	**0.000**
	nepalensis-ghunsa	0.022	0.010	0.034	**0.001**
	nepalensis-kanjiroba	-0.013	-0.028	0.002	0.086
HW: 6.62e-05***	*kanjiroba-ghunsa*	0.009	-0.021	0.039	0.729
	nepalensis-ghunsa	0.066	0.038	0.094	**0.000**
	nepalensis-kanjiroba	0.058	0.025	0.090	**0.001**
HL: 4e-07***	*kanjiroba-ghunsa*	0.031	0.009	0.053	**0.006**
	nepalensis-ghunsa	0.078	0.057	0.098	**0.000**
	nepalensis-kanjiroba	0.047	0.023	0.070	**0.000**
HW/HL: 0.0338*	*kanjiroba-ghunsa*	-0.123	-0.253	0.008	0.067
	nepalensis-ghunsa	-0.112	-0.233	0.010	0.074
	nepalensis-kanjiroba	0.011	-0.132	0.154	0.978
ED/HL: 0.0001***	*kanjiroba-ghunsa*	-0.109	-0.184	-0.034	**0.005**
	nepalensis-ghunsa	-0.154	-0.224	-0.085	**0.000**
	nepalensis-kanjiroba	-0.046	-0.128	0.037	0.343
IND: 0.00908**	*kanjiroba-ghunsa*	0.016	0.003	0.029	**0.014**
	nepalensis-ghunsa	0.012	0.000	0.024	**0.045**
	nepalensis-kanjiroba	-0.004	-0.018	0.010	0.744
FAL: 0.0291*	*kanjiroba-ghunsa*	0.005	-0.024	0.034	0.898
	nepalensis-ghunsa	0.031	0.004	0.058	**0.026**
	nepalensis-kanjiroba	0.026	-0.006	0.058	0.123
HAL: 1.54e-09***	*kanjiroba-ghunsa*	-0.214	-0.257	-0.171	**0.000**
	nepalensis-ghunsa	-0.194	-0.234	-0.154	**0.000**
	nepalensis-kanjiroba	0.020	-0.027	0.067	0.527
TIBL: 0.0117*	*kanjiroba-ghunsa*	0.010	-0.041	0.061	0.867
	nepalensis-ghunsa	0.063	0.015	0.110	**0.010**
	nepalensis-kanjiroba	0.053	-0.003	0.108	0.067
FOL: 0.0034**	*kanjiroba-ghunsa*	-0.009	-0.031	0.013	0.565
	nepalensis-ghunsa	0.027	0.006	0.047	**0.011**
	nepalensis-kanjiroba	0.036	0.011	0.060	**0.005**

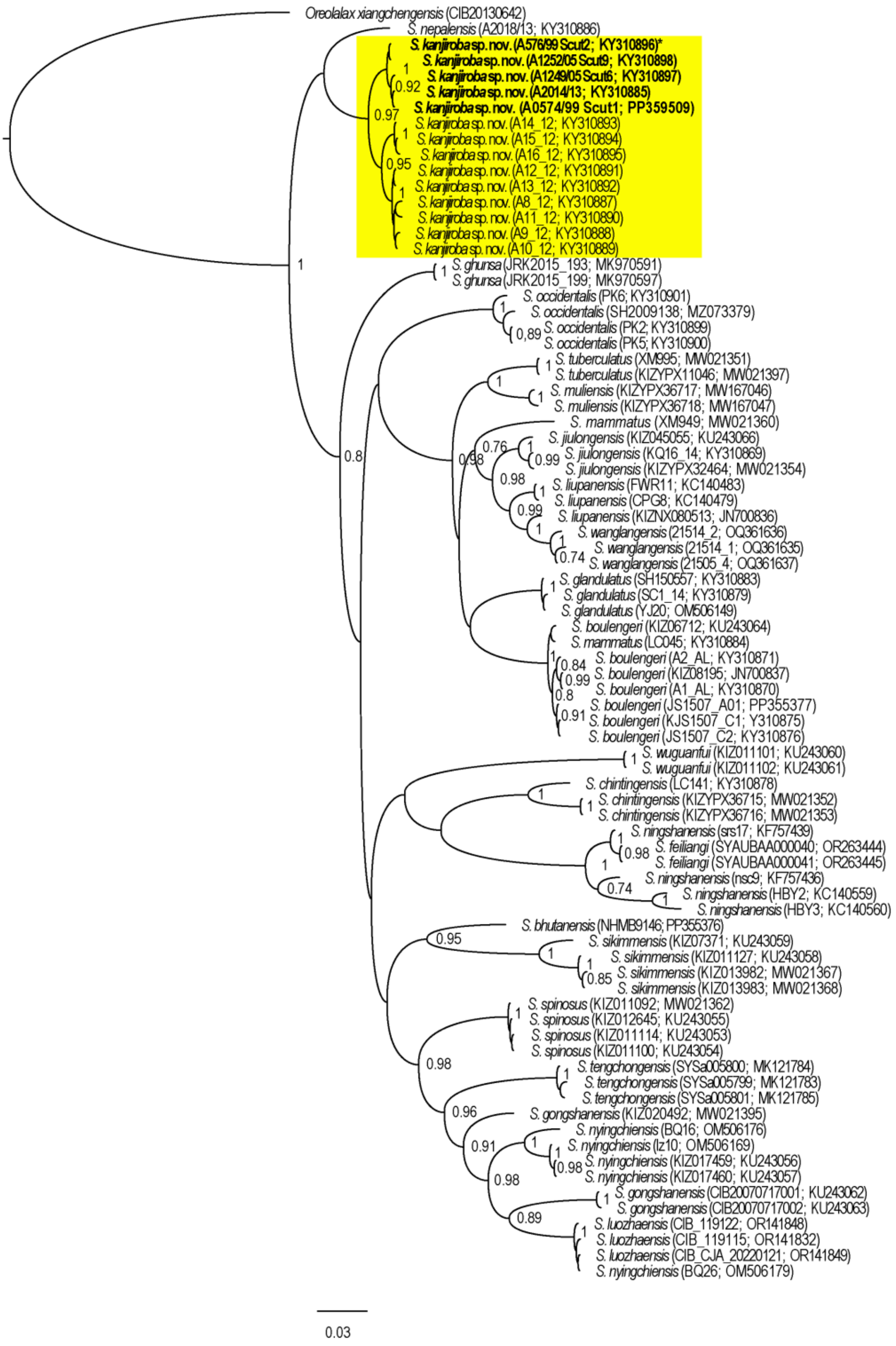

Fig. S1. Bayesian inference tree for the genus *Scutiger* based on coI mtDNA sequence data (631bp). Node values are Bayesian posterior probabilities ≥0.7. Species name is followed by voucher/sample ID and GenBank accession number. Holotype(*)/paratype labels are indicated bold.

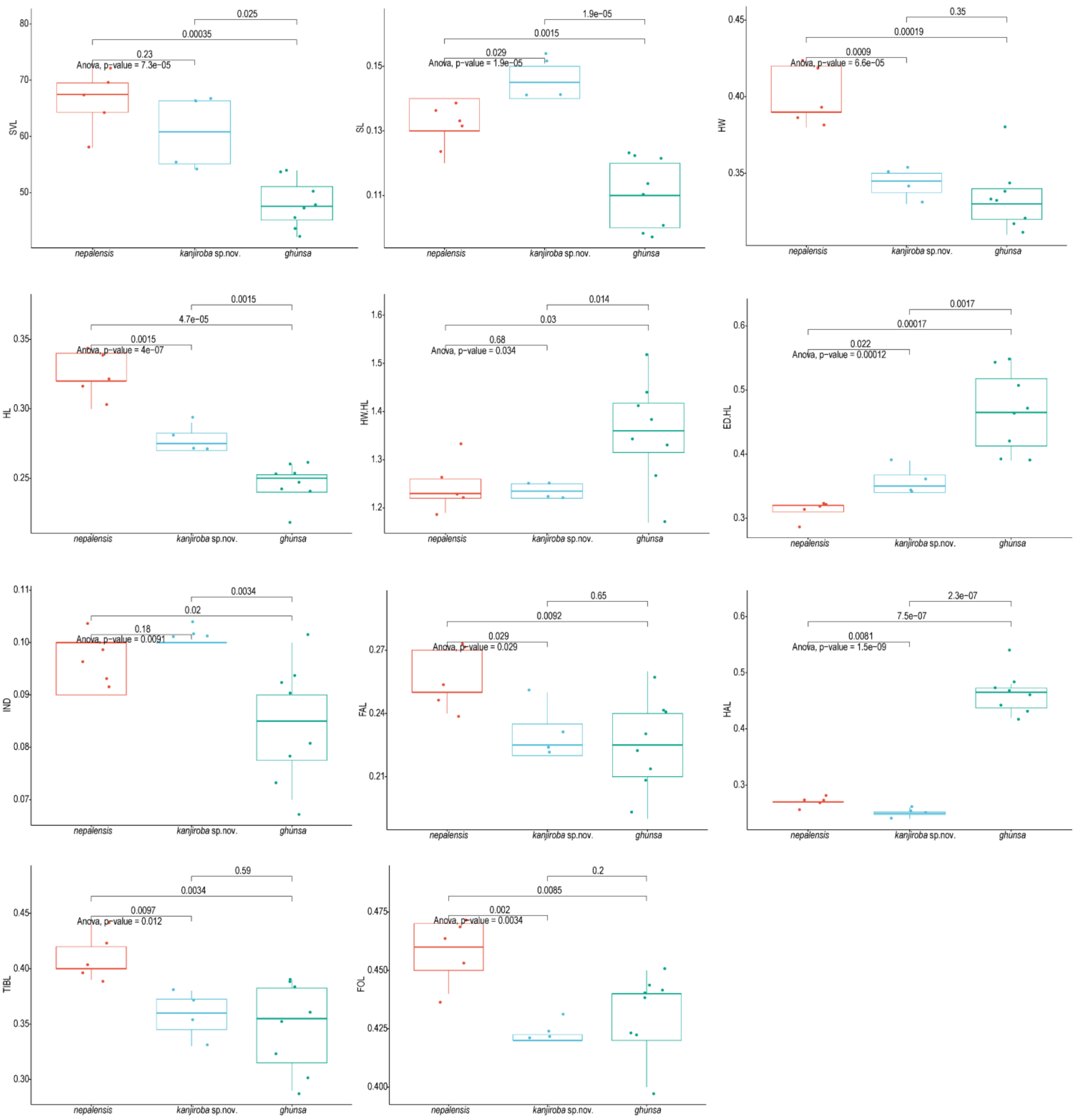

Fig. S2. Graphic representation of significant results of the one-way ANOVA for SVL and the different normalized morphometric characters and pairwise comparison between groups. SVL: Snout–vent length; SL: snout length; HW: head width; HL: head length; IND: internarial distance; FAL: forearm length; HAL: hand length; TIBL: tibia (shank) length; FOL: foot length.

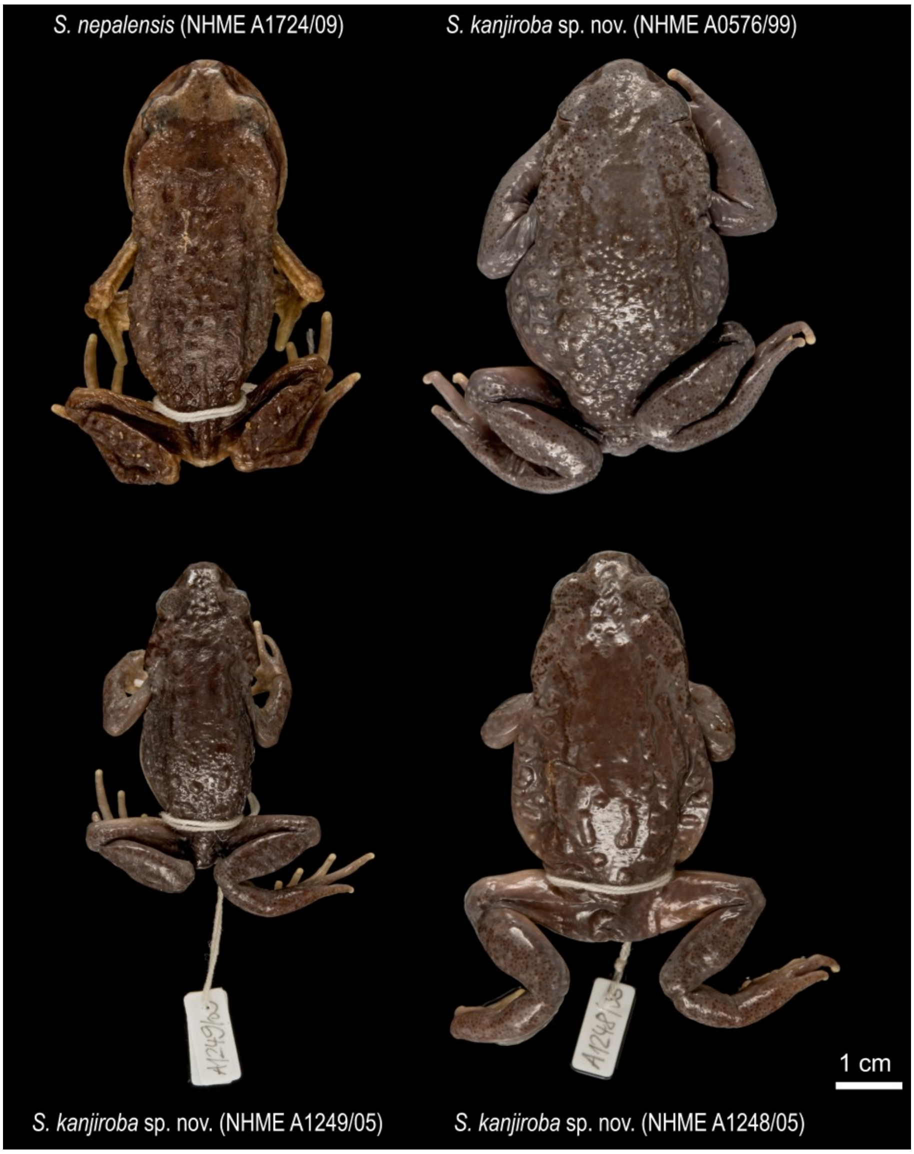

Fig. S3. Dorsal view of *Scutiger nepalensis* and *S. kanjiroba* **sp. nov.** see text for details (photo credits: M. Flecks). Notable, *S. nepalensis* has a considerable wider head shape than *S. kanjiroba* **sp. nov.**, and eyes of *S. kanjiroba* **sp. nov.** are more protruding from outline of head.

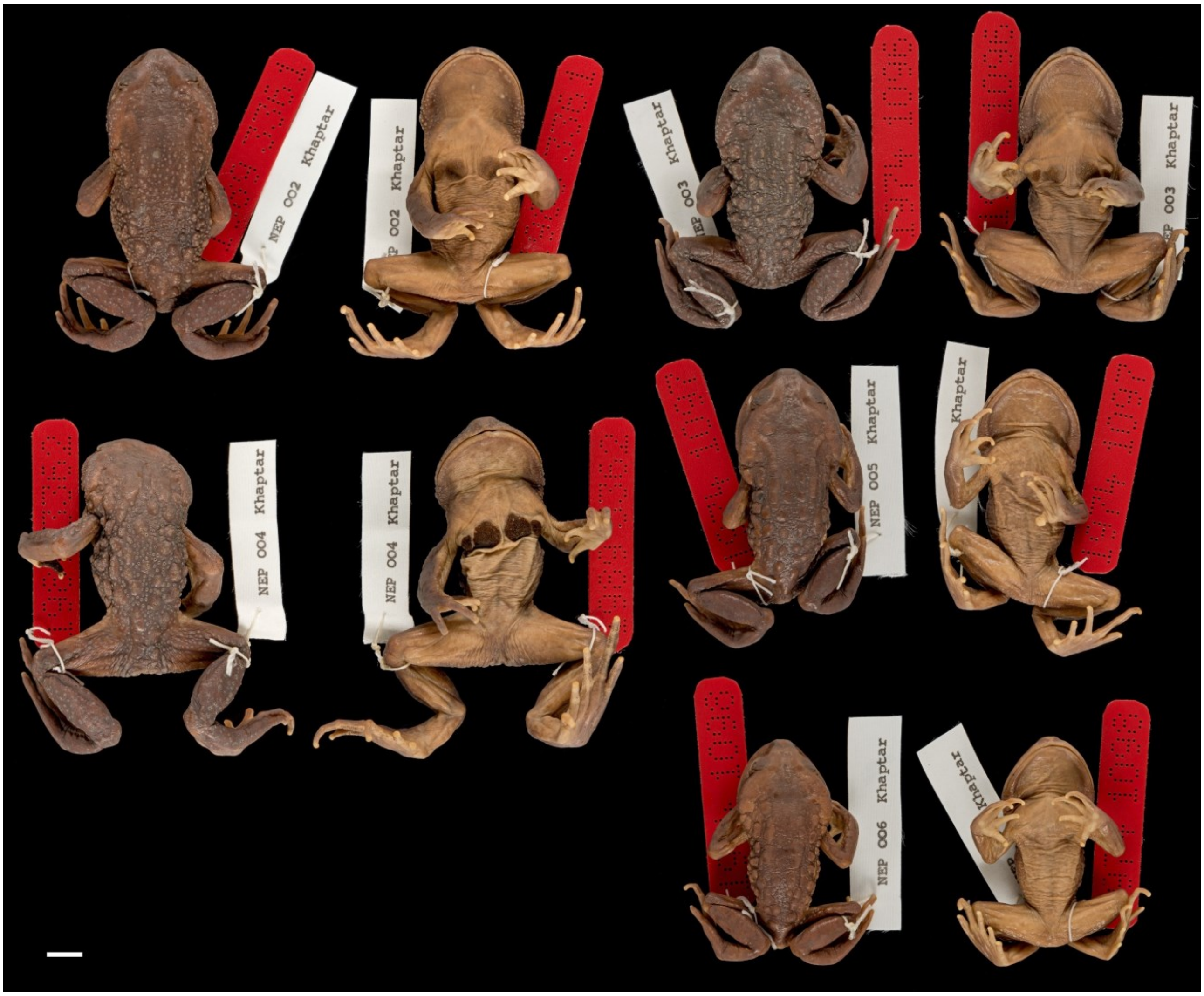

Fig. S4. Dorsal and ventral view of the paratype series of *Scutiger nepalensis* Dubois, 1974 (1974.1096–1974.1098 and 1989.3361–1989.3362) deposited at the MNMH (photo credits: M. Flecks). The scale bar corresponds to one centimeter.

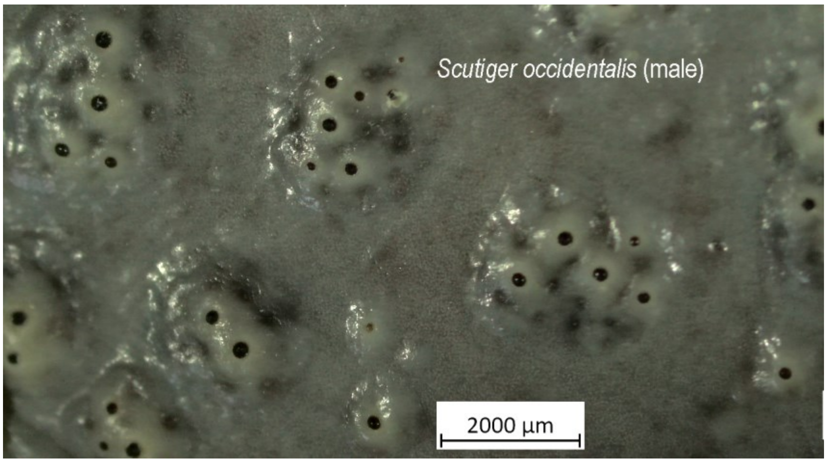

Fig. S5. Dorsal view of skin of adult male (ZFMK 103379) of *Scutiger occidentalis* from Lulusar, Pakistan, in non-breeding condition (photo credit: S. Hofmann)

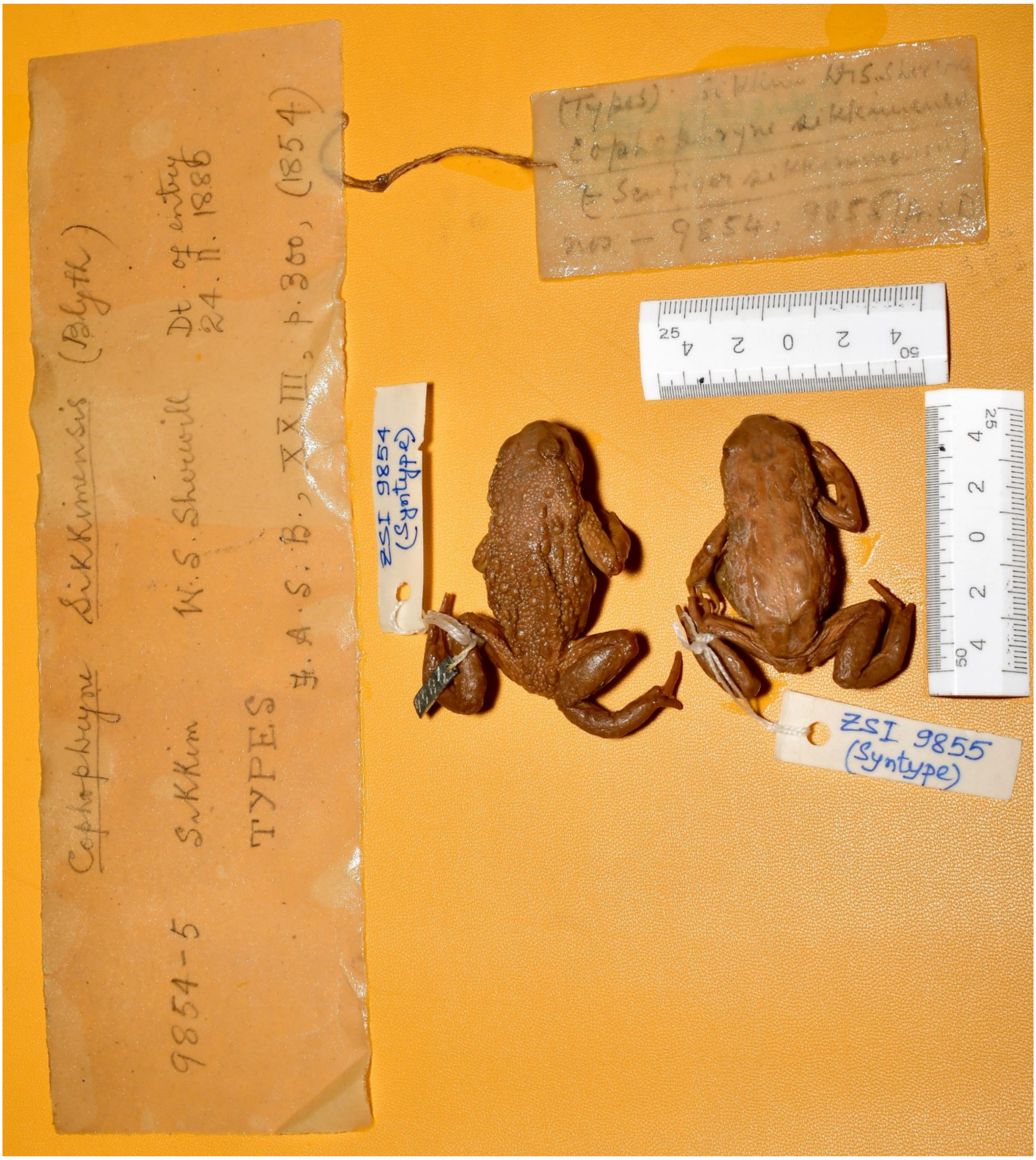

Fig. S6. Syntypes ZSIC 9854 and 9855 of *Scutiger sikimmensis* at the Zoological Survey of India, Kolkata (formerly Calcutta); credits: Pratyush P. Mohapatra.

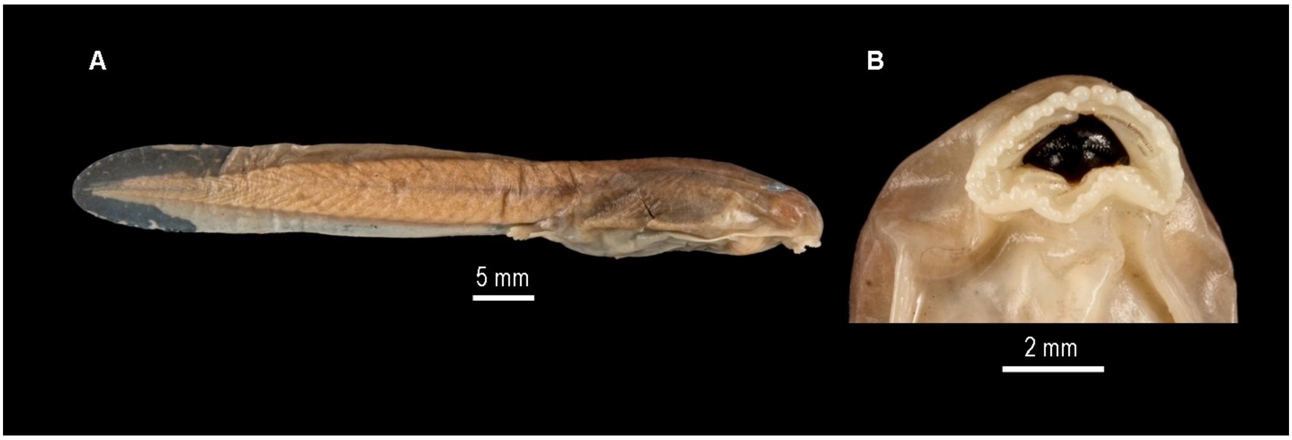

Fig. S7. Fixed tadpole at Gosner stage 36 of *Scutiger kanjiroba* **sp. nov.** (NHME A1250/05). A) lateral view; B) mouthpart (photo credits: M. Flecks).

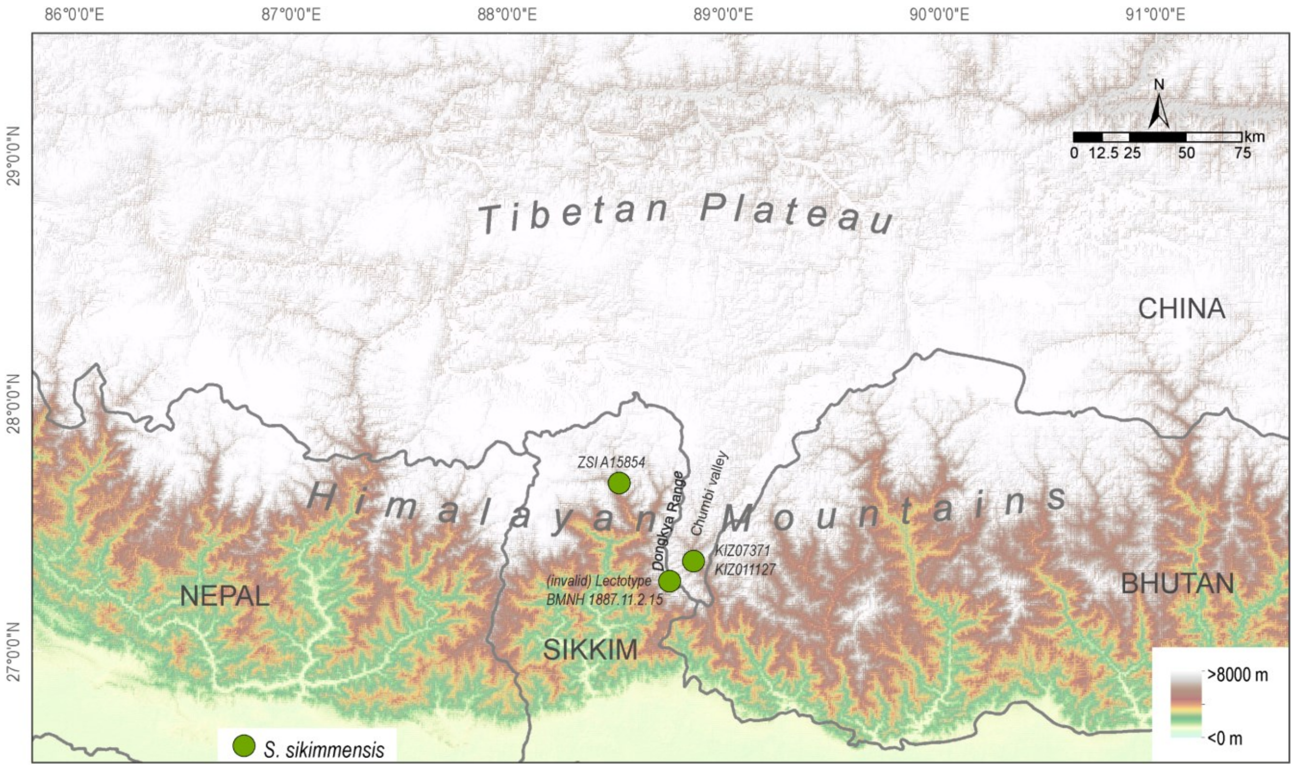

Fig. S8. Localities of *Scutiger sikimmensis*: BMNH 1887.11.2.15, invalid neotype from “Byutan, foot of Yakla Pass, Sikkim” (in eastern India), designated by Dubois (1987 "1986"); vouchers KIZ07371, KIZ011127, from Yadong, Tibet, China; voucher ZSI A15854 from Yumthang, North Sikkim, India, deposited at the Zoological Survey of India.

References

Bedriaga, J.d., 1898. Amphibien und Reptilien. Wissenschaftliche Resultate der von N. M. Przewalski nach Central-Asien unternommenen Reisen, & c./Nauchnuie Rezul'tatui puteshestvii N. M. Przheval'skagho po tzentral'noi Azii, & c. Volume 3, Zoologischer Theil, Part 1. Akadamie der Wissenschaften, St. Petersburg.

Blyth, E., 1855 "1854". Notices and descriptions of various reptiles, new or little known. Journal of the Asiatic Society of Bengal 23, 287–302.

Boulenger, G.A., 1987. On a rare Himalayan Toad, *Cophophryne sikkimensis*, Blyth. Annals and Magazine of Natural History, Series 5 20, 405–408.

Che, J., Jiang, K., Yan, F., Zhang, Y., 2020. Amphibians and Reptiles in Tibet – Diversity and Evolution. Chinese Academy of Sciences. Science Press, Beijing, China.

Das, I., Chanda, S.K., 2000. A new species of *Scutiger* (Anura: Megophryidae) from Nagaland, north-eastern India. Herpetological Journal London 10, 69–72.

Delorme, M., Dubois, A., 2001. Une nouvelle espèce de Scutiger du Bhutan, et quelques remarques sur la classification subgénérique du genre *Scutiger* (Megophryidae, Leptobrachiinae). Alytes 19, 141–153.

Dubois, A., 1974 "1973". Diagnoses de trois espèces nouvelles d'amphibiens du Népal. Bulletin de la Société Zoologique de France 98, 495–497.

Dubois, A., 1978. Une espèce nouvelle de *Scutiger* Theobald 1868 de l'Himalaya occidental (Anura: Pelobatidae). Senckenbergiana Biologica 59, 163-171.

Dubois, A., 1979. Une espèce nouvelle de *Scutiger* (Amphibiens, Anoures) du nord de la Birmanie. Revue Suisse de Zoologie 86, 631–640.

Dubois, A., 1987 "1986". Miscellanea taxinomica batrachologica (I). Alytes 5, 7–95.

Fang, R.-s., 1985. A new species of *Scutiger* from Shaanxi, China. Acta Herpetologica Sinica. New Series. Chengdu 4, 305–307 [In Chinese with English abstract].

Fei, L., 1977. A survey of amphibians in Xizang (Tibet). Acta Zoologica Sinica/ Dong wu xue bao. Beijing 23, 54–63.

Fei, L., Hu, S.-q., Huang, Y.-z., 2009. Fauna Sinica. Amphibia. Volume 2. Anura. Chinese Academy of Science. Science Press, Beijing.

Fei, L., Jiang, J.-p., Ye, C.-y., Cheng, S.-w., 1995. Electrophoresis analysis of crystalline lens protein of twenty one species (subspecies) of four genera of Pelobatidae. Acta Herpetologica Sinica, Chengdu 4–5, 230–237 [In Chinese with English abstract].

Fei, L., Ye, C.-y., 1986. A new species of the genus *Scutiger* from Hengduan Mountains (Amphibia: Pelobatidae). Acta Zoologica Sinica/ Dong wu xue bao. Beijing 32, 62–67 [In Chinese with English abstract].

Fei, L., Ye, C.J., Jiang, J.P., 2012. Colored Atlas of Chinese Amphibians and Their Distributions. Sichuan Science and Technology Press, Sichuan, China.

Günther, A.C.L.G., 1896. Report on the collections of reptiles, batrachians and fishes made by Messrs Potanin and Berezowski in the Chinese provinces Kansu and Sze-chuen. .Annuaire du Musée Zoologique de l'Academie Impériale des Sciences de St. Pétersbourg 1, 199-219.

Huang, Y.-z., 1985. A new species of pelobatid toads (Amphibia: Pelobatidae) from Ningxia Hui Autonomous Region. Acta Biologica Plateau Sinica 4, 77–81.

Jiang, K., Rao, D.-Q., Yuan, S.-Q., Wang, J.-S., Li, P.-P., Hou, M., Che, M., Che, J., 2012. A new species of the genus *Scutiger* (Anura: Megophryidae) from southeastern Tibet, China. Zootaxa 3388, 29–40.

Jiang, K., Wang, K., Zou, D.-H., Yan, F., Li, P.-P., Che, J., 2016. A new species of the genus *Scutiger* (Anura: Megophryidae) from Medog of southeastern Tibet, China. Zoological Research 37, 21-30.

Khatiwada, J.R., Shu, G.C., Subedi, T.R., Wang, B., Ohler, A., Canatella, D.C., Xie, F., Jiang, J.P., 2019. A new species of megophryid frog of the genus *Scutiger* from Kangchenjunga Conservation Area, eastern Nepal. Asian Herpetological Research 10, 139–157.

Liu, C.-C., 1950. Amphibians of western China. Fieldiana. Zoology Memoires 2, 1–397.

Liu, C.-c., Fei, L., 1979. Five new pelobatid toads from China. Acta Zootaxonomica Sinica/ Dong wu fen lei xue bao. Beijing 4, 83–92.

Liu, C.-c., Hu, S.-q., Tian, W.-s., Wu, G.-f., 1978. Four new species of amphibians from Sichuna and Guangxi [In Chinese]. Materials for Herpetological Research. Chengdu 4, 18-19

Rao, D., 2022 "2020". Atlas of Wildlife in Southwest China: Amphibian [in Chinese]. In: Zhu, J.-G., Rao, D. (Eds.), Atlas of Wildlife in Southwest China: Amphibian (printed in 2020 but not distributed until 2022). Beijing Publishing Group, Beijing, pp. 1–448.

Shi, S., Sui, L.-L., Ma, S., Ji, F.-R., Bu-Dian, A.-Y., Jiang, J.-p., 2023. A new Asian lazy toad of the genus *Scutiger* Theobald, 1868 (Anura, Megophryidae) from southern Tibet, China. ZooKeys 1187, 31–62.

Yang, D.-t., Su, C.-y., Li, S.-m., 1979. New species and new subspecies of amphibians and reptiles from Gaoligong Shan, Yunnan. Acta Zootaxonomica Sinica/ Dong wu fen lei xue bao. Beijing 4, 185–188.
Yang, J.-h., Huang, X.-y., 2019. A new species of *Scutiger* (Anura: Megophryidae) from the Gaoligongshan Mountain Range, China. Copeia 107, 10-21.
Ye, C.-y., Fei, L., 2007. A new species of Megophryidae—*Scutiger* (*Scutiger*) *wanglangensis* from Sichuan, China (Amphibia, Anura). Herpetologica Sinica/Liang qi pa xing dong wu xue yan jiu 11, 33–37.
Zhou, S., Guan, P., Shi, J., 2023. A new species of the genus *Scutiger* from eastern Qinling Mountains (Anura: Megophryidae) [In Chinese with English abstract]. Chinese Journal of Zoology 58, 641–650.
